# Benevolent Childhood Experiences and Suicidal Ideation Among College Students: A Moderation Analysis by Age, Gender, and Marital Status

**DOI:** 10.3390/healthcare14213574

**Published:** 2026-10-22

**Authors:** Haydee Mercedes Aguilar-Armas, Velia Graciela Vera-Calmet, Marco Agustín Arbulú Ballesteros, Olga Elizabeth Samanamud Ramírez, Milagros del Carmen Quispe Villarreal, Lucy Angélica Yglesias-Alva

**Affiliations:** 1 Institute for Research in Science and Technology, Chepén Campus, César Vallejo University, Trujillo 13001, Peru; haguilarar@ucvvirtual.edu.pe (H.M.A.-A.); vverac@ucv.edu.pe (V.G.V.-C.); samanamud@ucvvirtual.edu.pe (O.E.S.R.); mquispevi@ucv.edu.pe (M.d.C.Q.V.); 2 Department of Statistics, School of Physical and Mathematical Sciences, National University of Trujillo, Trujillo 13001, Peru; lyglesias@unitru.edu.pe

**Keywords:** protective factors, mental health, suicide, emerging adulthood, multiple regression, higher education, Peru

## Abstract

**Highlights:**

**What are the main findings?**

- Benevolent childhood experiences were inversely and significantly associated with the frequency of suicidal ideation in 451 Peruvian college students (*r* = −0.358, *p* < 0.001).
- The inverse association weakened with age (continuous-age interaction *b* = 0.061, 95% CI [0.026, 0.097]; Δ*R*^2^ = 0.020) and was no longer statistically significant beyond approximately 31 years of age (Johnson–Neyman), whereas gender and marital-status interactions were not significant; the age result is presented as exploratory given the small number of older participants.

**What are the implications of the main findings?**

- The inverse association between childhood protective capital and suicidal ideation did not differ significantly by gender or marital status, although the power to detect small interactions was limited, so equivalence across groups cannot be claimed.
- The Spanish BCEs scale showed acceptable unidimensional fit (WLSMV CFA) and a marginal KR-20; its use should remain confined to research until a formal Peruvian validation is available, and screening applications are not recommended at this stage.

**Abstract:**

**Background/Objectives**: Suicidal ideation affects a considerable proportion of college students, and protective factors developed during childhood may mitigate it. We examined the relationship between benevolent childhood experiences (BCEs) and the frequency of suicidal ideation among Peruvian college students, as well as the moderating roles of age, gender, and marital status. **Methods**: A total of 451 students from public and private universities in Peru (74.9% women; mean age 22.12 years) participated in the study and completed the BCEs scale and the Spanish version of the Suicidal Ideation Frequency Inventory. Using a cross-sectional correlational design, three moderation models were estimated via multiple linear regression with mean-centered predictors, heteroscedasticity-consistent (HC3) and bootstrap confidence intervals, and sensitivity analyses. **Results**: BCEs were inversely and significantly associated with suicidal ideation (*r* = −0.358, *p* < 0.001). Age moderated this relationship: in the continuous-age model, the interaction was *b* = 0.061 (95% CI [0.026, 0.097]; Δ*R*^2^ = 0.020), and the inverse association was no longer significant beyond approximately 31 years of age, a result presented here as exploratory because it rests partly on a small number of older students. Gender and marital-status interactions were not significant, although the power to detect small interactions was limited; the models explained between 14% and 21% of the variance. **Conclusions**: The findings indicate that benevolent childhood experiences are inversely associated with suicidal ideation, an association that did not differ significantly by gender or marital status and that appears strongest in emerging adulthood. These findings are correlational and do not establish a protective effect; the assessment of BCEs merits further study in university mental health research, pending validation of the scale in the Peruvian population.

## 1. Introduction

Suicidal ideation encompasses thoughts, cognitions, and plans related to the act of taking one’s own life and is the strongest predictor of suicide attempts and completed suicide [1,2]. Among the university population, the transition to higher education triggers a period of high psychosocial vulnerability characterized by simultaneous exposure to academic stressors, the reorganization of emotional bonds, and uncertainty regarding career and identity [3,4]. Mortier et al. [3] estimated that the prevalence of suicidal ideation among college students reaches 22.3% over a lifetime and 10.6% in the past 12 months, with substantial variations depending on cultural context, gender, and the presence of psychopathology.

According to the World Health Organization (WHO) [5], suicidal ideation is one of the most relevant mental health indicators in the field of public health, and suicide is the fourth leading cause of death among people aged 15 to 29. It is estimated that for every completed suicide, there are between 10 and 20 prior nonfatal attempts, which highlight the magnitude of the phenomenon and the urgency of identifying risk and protective factors [2,5].

To understand how this phenomenon arises, Joiner’s psychological interpersonal model of suicide [6] proposes that suicidal ideation emerges when two psychological states converge: frustrated belonging—a sense of not being meaningfully connected to others—and the perception of being a burden to one’s environment [2,7,8]. Both states intensify in the face of loss, rejection, or failure during critical periods of development. Positive experiences act as structural support by providing cognitive-relational schemas of self-worth, identity, and hope. Emotional Regulation Theory [9] posits that individuals who had secure caregivers and school environments during childhood develop adaptive strategies for affect regulation, reducing the likelihood that college-related stressors will trigger suicidal ideation.

Among these early resources, benevolent childhood experiences (BCEs) encompass the protective resources acquired during childhood. They include positive experiences, such as the presence of secure caregivers, stable friendships, support from significant adults outside the home, reassuring beliefs, and the availability of predictable household routines [10]. It has been demonstrated that higher levels of BCEs are associated with lower levels of perceived adversity, greater psychological resilience, and a reduced risk of psychopathology in adulthood [11,12,13]. Bethell et al. [14] have proposed that resilience functions within BCEs as a biopsychosocial buffer, modulating the negative impact of early adversity on mental health outcomes throughout the lifespan. BCEs are consistent with Bronfenbrenner’s ecological systems model [15], in which positive microsystems support socioemotional development and modulate vulnerability to subsequent stressors.

In the specific area of suicide risk, Akbulut [16] and Kallapiran et al. [17] have provided evidence of the association between benevolent childhood experiences and lower levels of suicidal ideation and behavior in adolescents and young adults [18,19]. These findings position benevolent childhood experiences as a highly valuable construct for preventive research in college mental health.

Despite these advances, studies examining the association between benevolent childhood experiences and suicidal ideation among college students are scarce, especially in Latin America [7,8,20,21]. These studies focus on clinical samples or adolescents in developed countries, leaving a considerable gap in populations with sociodemographic diversity [22,23]. The prevalence of suicidal ideation varies significantly depending on gender, socioeconomic status, and the presence of stressful life events [24,25,26].

This gap also extends to the role of sociodemographic moderators. Age has a solid theoretical basis as a moderator; the early adult years (ages 16–25) are characterized by greater emotional lability and identity exploration processes that can amplify both the protective and risk effects of previous experiences [27,28]. Gender has been identified as a moderating variable in mental health studies; women have a higher prevalence of suicidal ideation, while men report a higher risk of completed suicide attempts, suggesting differences in symptomatic expression and coping [29,30,31,32]. Marital status is associated with perceived social support networks and the burden of responsibilities—factors that, in interaction with early life experiences, may influence vulnerability to suicidal ideation [2,3,33].

Considering the above, the general objective of this study is to examine the relationship between benevolent childhood experiences and the frequency of suicidal ideation in a sample of Peruvian college students, and to analyze whether this relationship varies according to age, gender, and marital status as sociodemographic moderators. The specific objectives are to determine the magnitude and direction of the association between benevolent childhood experiences and suicidal ideation; and to test moderation models for each sociodemographic variable to identify groups that are differentially protected or vulnerable. This study contributes to the body of evidence on early protective factors in college mental health in Latin America and offers concrete implications for the design of targeted preventive interventions [24,26,34].

### 1.1. Study Hypotheses

#### 1.1.1. General Hypothesis

**GH.**  *Benevolent childhood experiences are inversely and significantly associated with the frequency of suicidal ideation among Peruvian college students, and this association varies according to sociodemographic moderators (age, gender, and marital status).*

#### 1.1.2. Hypotheses on the Main Relationship Between Benevolent Childhood Experiences and Suicidal Ideation

**H1.**  *Benevolent childhood experiences are negatively and significantly associated with the frequency of suicidal ideation among Peruvian college students, such that the greater the accumulation of protective childhood experiences, the lower the frequency of suicidal thoughts [14,16,17].*

**H2.**  *Age moderates the relationship between benevolent childhood experiences and the frequency of suicidal ideation, such that the inverse association between BCEs and suicidal ideation is more pronounced in the young adult group (ages 16–25) than in older age groups [11,27,28].*

**H3.**  *Gender moderates the relationship between benevolent childhood experiences and the frequency of suicidal ideation, such that the inverse association between BCEs and suicidal ideation differs between women and men [16,18,30,35].*

**H4.**  *Marital status moderates the relationship between benevolent childhood experiences and the frequency of suicidal ideation, with the inverse association being most pronounced among single college students, for whom the psychosocial capital internalized during childhood would compensate for the lack of support from a partner [2,6,36].*

## 2. Materials and Methods

### 2.1. Study Design and Type

The study adopted a non-experimental cross-sectional design with a correlational scope. A multiple moderation analysis model was employed to examine whether the association between benevolent childhood experiences (BCEs) and the frequency of suicidal ideation (FIS) varies according to sociodemographic variables (age, gender, and marital status) [37,38,39]. The cross-sectional design implies that all measurements were taken at a single point in time, which allows for the establishment of statistical associations [40,41].

### 2.2. Participants and Sampling Procedure

The final sample consisted of 451 college students from public and private universities in Peru, mostly from institutions in the La Libertad and Áncash regions. The age range was between 17 and 66 years (*M* = 22.12, *SD* = 6.23), with a predominance of the emerging adulthood group aged 16 to 25 (82.3%). The sample was predominantly female (74.9%), unmarried (90.2%), and resided in an urban setting (80.9%). Regarding their field of study, 69.0% were majoring in psychology, followed by business administration (16.0%) and other disciplines.

A non-probabilistic convenience sampling method was used [42,43]. The inclusion criteria required participants to be college students enrolled in the 2025-II semester who had provided informed consent; for the 20 participants under the age of 18, parental or guardian consent and informed assent were also required. Of the 485 responses received, 2 questionnaires with omissions exceeding 10% of the items were excluded. Because the form recorded each respondent’s e-mail address, repeated submissions could be identified; 28 participants had submitted the questionnaire two or three times (60 submissions in total), presumably by reopening the form, so only the first submission of each participant was retained and the remaining 32 records were removed, resulting in a final sample of 451 cases. No item-level missing data remained in the retained questionnaires, so no imputation was required; the seven cases with a missing university cycle were retained for all analyses that did not involve that variable.

**Procedure.** Data were collected between November and December 2025 (second semester of the 2025 academic year), once ethics approval on 10 November 2025 had been obtained and before any participant was recruited. Recruitment took place in the classroom: members of the research team visited each participating class, explained the objectives of the study, the voluntary nature of participation, and the sensitive content of some items, and invited the students present to complete a self-administered online questionnaire (Google Forms; Google LLC, Mountain View, CA, USA) on their own devices in a single session. No incentives were offered. According to the attendance records of the participating classes, 455 students were present during the visits; 453 of them completed the questionnaire (response rate = 99.6%; 485 submissions were received because 28 participants submitted the form more than once, see above). After excluding two questionnaires with omissions exceeding 10% of the items, the final analytical sample comprised 451 students (99.1% of those present). The form collected the respondent’s institutional or personal e-mail address, which was used to detect and remove repeated submissions (see above); the address was deleted from the analytical dataset once duplicates had been screened. Informed consent (and parental consent plus assent for minors) was obtained on the first page of the form. Because the questionnaire included items on suicidal ideation, a safety protocol was agreed in advance with the psychological service of each participating university: any participant who disclosed suicidal ideation to the research team, requested help, or was identified by the team as requiring attention during or after the session would be referred to that service—no participant requested assistance or was referred during data collection. Information on mental health resources was presented on the final page of the questionnaire, after the items, so that the information itself did not prime the responses; the consent page had already informed participants of the sensitive content and of the availability of support, and the team remained in the classroom while the questionnaire was completed.

The sample was predominantly female, young, single, and urban, with a significant proportion of participants who were not employed and were enrolled in the early stages of their programs (see Table 1).

### 2.3. Instruments

**Benevolent Childhood Experiences Scale (BCEs).** Throughout this article the construct is designated benevolent childhood experiences (BCEs), in accordance with the instrument administered; the literature uses this label interchangeably with positive childhood experiences (PCEs), and both terms are routinely searched as synonyms in systematic reviews of the field. The Benevolent Childhood Experiences Scale (BCEs) [10] was administered using the Spanish version that was made available by the developers of the scale; no new translation was undertaken by the research team, and the wording of the ten items was used verbatim. The scale consists of 10 dichotomous response items (0 = No/1 = Yes) that assess the cumulative presence of protective experiences during childhood, such as the availability of a secure caregiver, stable friendships, and access to predictable daily routines. The total score ranges from 0 to 10, with higher values indicating greater childhood protective capital. Because the Spanish version has not been validated in Peru, its psychometric performance was examined in the present sample and is reported in full in the Supplementary Materials (Section S1). In brief, KR-20 was 0.66, a marginal value that is nevertheless expected for a short cumulative index of heterogeneous experiences [44]; the ordinal alpha based on tetrachoric correlations was 0.82. A one-factor confirmatory factor analysis (CFA) on the tetrachoric correlation matrix with the WLSMV estimator showed acceptable fit (scaled *χ*^2^(35) = 58.04, *p* = 0.009; CFI = 0.955; TLI = 0.942; RMSEA = 0.038, 90% CI [0.019, 0.055]; SRMR = 0.082), with standardized loadings between 0.32 and 0.75 and a categorical omega of 0.82. A Rasch (one-parameter logistic) model yielded item difficulties ranging from −1.02 (item 4, “I liked school”) to 0.93 (item 10, “predictable daily routine”), with infit mean square values between 0.81 and 1.13 (Table S3); the Martin-Löf test did not reject unidimensionality (*p* = 0.147), whereas the Andersen likelihood-ratio test indicated some departure from parameter invariance across score levels (*p* < 0.001 for the mean split), and person-separation reliability was low (0.31), as expected for a 10-item dichotomous index with a strong ceiling. Evidence based on relations with other variables was consistent with theory: BCEs correlated negatively with the FIS score (Spearman *ρ* = −0.32, *p* < 0.001) and were lower among students who reported having thought about suicide at least “sometimes” in item 4 of the FSII (Mann–Whitney *U* = 25,923, *p* < 0.001, *r* = 0.32), whereas they did not differ by gender, residence area, employment, or age group (Table S4). No other established scale was administered, so convergent validity with an external instrument could not be examined and is noted as a limitation.

**Suicidal Ideation Frequency Inventory (FSII).** Suicidal ideation was measured using the Suicidal Ideation Frequency Inventory (FSII) [45], a five-item scale with a five-point Likert-type response format (1 = never to 5 = always) and scores ranging from 5 to 25, in its Spanish version [46]. The scale demonstrated excellent internal consistency (Cronbach’s α = 0.94; ordinal α = 0.96) and corrected item-total correlations ranging from 0.810 to 0.872. A one-factor CFA with the WLSMV estimator on the polychoric correlation matrix showed standardized loadings between 0.90 and 0.94, categorical omega = 0.96, and average variance extracted (AVE) = 0.83 (scaled *χ*^2^(5) = 33.94, *p* < 0.001; CFI = 0.998; TLI = 0.996; RMSEA = 0.113, 90% CI [0.079, 0.151]; SRMR = 0.013); the elevated RMSEA reflects the very low degrees of freedom of a five-item model rather than substantive misfit [47]. The empirical distinctness of the two measures was examined with a two-factor CFA (BCEs and FSII items, WLSMV), which fitted well (CFI = 0.993; TLI = 0.992; RMSEA = 0.047, 90% CI [0.037, 0.057]; SRMR = 0.084) and significantly better than a single-factor model with all fifteen items (Satorra–Bentler scaled Δ*χ*^2^(1) = 82.90, *p* < 0.001); the latent correlation between the factors was −0.53 (95% CI [−0.62, −0.44]), indicating related but distinguishable constructs. Hereafter, FSII designates the measurement instrument by its original name, while BCEs and FIS refer to the constructs and their scores; the same convention is applied to all tables.

The descriptive statistics show that benevolent childhood experiences are concentrated in the upper part of the scale (*M* = 7.92 on a 0–10 range; 23.9% of participants endorsed all ten items), whereas the FIS score is positively skewed (skewness = 0.73) with a floor effect (30.2% of participants at the minimum score of 5). In the descriptive comparison by gender, women report a higher mean score for suicidal ideation than men, although this observation should not be interpreted as inferential evidence on its own. At the item level, the most frequent positive experiences are related to enjoying school, the opportunity to have fun, and the presence of protective relationships, whereas on the FSII, the highest average scores are associated with thoughts about ending one’s life and the perception of not deserving to live (see Table 2).

The results show that both scales function adequately for research purposes. The BCEs scale exhibits marginal reliability in classical terms (KR-20 = 0.66), which is consistent with its nature as a cumulative index of heterogeneous experiences and should be considered when interpreting effect sizes. The FSII exhibits adequate internal consistency and high item-total correlations, evidence that its items converge homogeneously toward a single construct. The weak-to-acceptable discrimination indices of BCEs reflect the very nature of the construct: benevolent childhood experiences are, by definition, diverse and do not necessarily converge with one another (see Table 3).

The indicators presented in Table 4 support the use of both measures in the present study. The FSII behaves as a strongly unidimensional and highly reliable scale. The BCEs index shows acceptable unidimensional fit with moderate loadings, in line with its conception as a cumulative index rather than a reflective construct, and its classical reliability should be regarded as marginal. The two-factor model fits substantially better than a single common factor, and the latent correlation of −0.53 indicates that BCEs and suicidal ideation are related but empirically distinguishable, a prerequisite for interpreting the moderation models.

### 2.4. Data Analysis

The analysis was organized into three stages. First, descriptive statistics and distribution indices were calculated to characterize the sample and describe the behavior of the study variables. The psychometric properties of the BCEs and the FSII were examined with classical indices (KR-20, Cronbach’s α, ordinal α, corrected item-total correlations, mean inter-item correlation) and with confirmatory factor analyses estimated with WLSMV on tetrachoric/polychoric correlation matrices, from which categorical omega and AVE were derived; a Rasch (one-parameter logistic) model was fitted to the BCEs items, and known-groups comparisons were conducted with Spearman correlations and Mann–Whitney U tests [47,52]. Scale scores were computed as the unweighted sum of item responses (BCEs: 0–10; FIS: 5–25); as no item-level data were missing in the retained cases, no imputation or proration was needed. Three independent moderation models were estimated for each sociodemographic variable—age, gender, and marital status—using multiple linear regression with an interaction term (BCEs × moderator) [37]. BCEs scores were mean-centered (*M* = 7.92) before the products were formed, so that the coefficients of the moderators express group differences at the average level of BCEs.

Age was entered as a multi-categorical variable using two categories (26–35 years and 36 years or older; reference group: 16–25 years). The cut-offs were specified a priori on theoretical grounds, before the data were analyzed, following the developmental distinction between emerging adulthood (up to 25 years) [27], which characterizes the vast majority of the sample, and established adulthood (26–35 years), with a residual category of students aged 36 years or older; they were not derived from the data, and the categories are labeled by their age ranges rather than by developmental-stage labels. Because the oldest group is small (*n* = 21), the categorical model is complemented by a model in which age is treated as a mean-centered continuous variable, which uses all the information in the data and does not depend on the cut-offs. Gender was analyzed using a dummy variable (0 = female, 1 = male), excluding from the inferential analysis those who chose not to disclose it (*n* = 5) due to insufficient sample size, and marital status was dichotomized (0 = single, 1 = not single); because the non-single category combines heterogeneous situations (cohabiting, married, divorced, and widowed), a sensitivity analysis compared partnered students (cohabiting or married, *n* = 41) with single students after excluding the three divorced or widowed participants, and the cohabiting and married subgroups were also examined separately. For each model, the unstandardized coefficients (B), standard error (SE), *t*-statistic, *p*-value, 95% confidence intervals, and the change in *R*^2^ (ΔR^2^) attributable to the interaction term are reported, together with simple slopes and their confidence intervals for each level of the moderator; for the continuous-age model, the region of significance was identified with the Johnson–Neyman technique. Because the FIS score is bounded and positively skewed, regression assumptions were checked for every model: the distribution of standardized residuals (skewness, kurtosis, Shapiro–Wilk test), heteroscedasticity (Breusch–Pagan and White tests), influential observations (Cook’s distance, leverage, DFBETAS), multicollinearity (variance inflation factors), and linearity of the BCEs–FIS relation (quadratic BCEs term and Ramsey RESET test) (Table S5). Since heteroscedasticity was detected in all models, heteroscedasticity-consistent (HC3) standard errors and bias-corrected and accelerated (BCa) bootstrap confidence intervals based on 5000 case resamples are reported alongside the ordinary least squares estimates (Table S6). Sensitivity analyses re-estimated each model after excluding observations with Cook’s distance greater than 4/n, with a log-transformed outcome, with MM-type robust regression, and, for the age model, after excluding participants older than 50 years and participants aged 36 years or older (Table S7). As an additional sensitivity analysis, each model was re-estimated adjusting for the covariates available in the dataset (university, field of study, residence area, and employment status); no measures of depressive symptoms, anxiety, adverse childhood experiences, current distress, substance use, socioeconomic status, or social support were collected, so adjustment for these confounders was not possible (Table S9; see Limitations). No a priori power analysis was conducted: the sample size was determined by the number of classes to which access was granted. Post hoc sensitivity analyses indicate that *N* = 451 provides 80% power (α = 0.05, two-sided) to detect a bivariate correlation of |r| ≥ 0.13 and interaction effects of *f*^2^ ≥ 0.018 (Δ*R*^2^ ≈ 0.015); the observed f^2^ of each interaction is reported for comparison (Table S8). All analyses were conducted at a significance level of α = 0.05. No adjustment for multiple testing was planned a priori, because H2–H4 were formulated as three separate, theoretically motivated hypotheses; nevertheless, because three interaction tests were performed, Bonferroni- and Holm-adjusted *p*-values are reported alongside the unadjusted ones (family-wise threshold α = 0.017), and a moderation finding is described as confirmatory only if it meets the adjusted criterion, and otherwise as exploratory. Descriptive analyses and the original moderation models were performed in SPSS version 28.0 (IBM Corp., Armonk, NY, USA) [53] with the PROCESS macro [37] and in JASP 0.18 (JASP Team, Amsterdam, The Netherlands) [54]; the confirmatory factor, Rasch, diagnostic, robust, and bootstrap analyses added during revision were performed in R 4.3.3 (R Foundation for Statistical Computing, Vienna, Austria) with the packages lavaan, eRm, psych, sandwich, lmtest, car, MASS, and boot.

### 2.5. Ethical Considerations

The study was conducted in full accordance with the ethical principles of the Declaration of Helsinki [55] and with the Code of Ethics and Professional Conduct of the Peruvian College of Psychologists [56]. Since the assessment included items related to suicidal ideation, a safety protocol was implemented in accordance with recommendations for research involving vulnerable populations [57]: participants were informed of the sensitive content before consenting, the research team remained present during administration, a referral pathway to the psychological service of each university was agreed in advance for any participant who disclosed ideation or requested help (none did), and information on mental health resources was provided at the end of the questionnaire so as not to prime the responses (see Section 2.2). The data were stored in encrypted form, and only the research team had access to the database, always ensuring confidentiality [58]. This research was reviewed and approved by the Research Ethics Committee of the School of Psychology at César Vallejo University under Report No. 01807-2025/CEI-PSI on 10 November 2025; recruitment and data collection took place between November and December 2025 once approval had been granted.

## 3. Results

The bivariate association between BCEs and FIS was negative and significant (*r* = −0.358, 95% CI [−0.436, −0.275], *p* < 0.001; Spearman *ρ* = −0.32; unstandardized slope *b* = −0.94 FIS points per benevolent experience, i.e., 0.18 SD), supporting H1: the greater the accumulation of protective childhood experiences, the lower the frequency of suicidal ideation. The three moderation models are presented below; all coefficients refer to mean-centered BCEs, and Tables S5–S8 report the diagnostics, robust and bootstrap intervals, sensitivity analyses, and simple slopes for each model.

### 3.1. Model 1. Age as a Moderator

**Figure 1:**
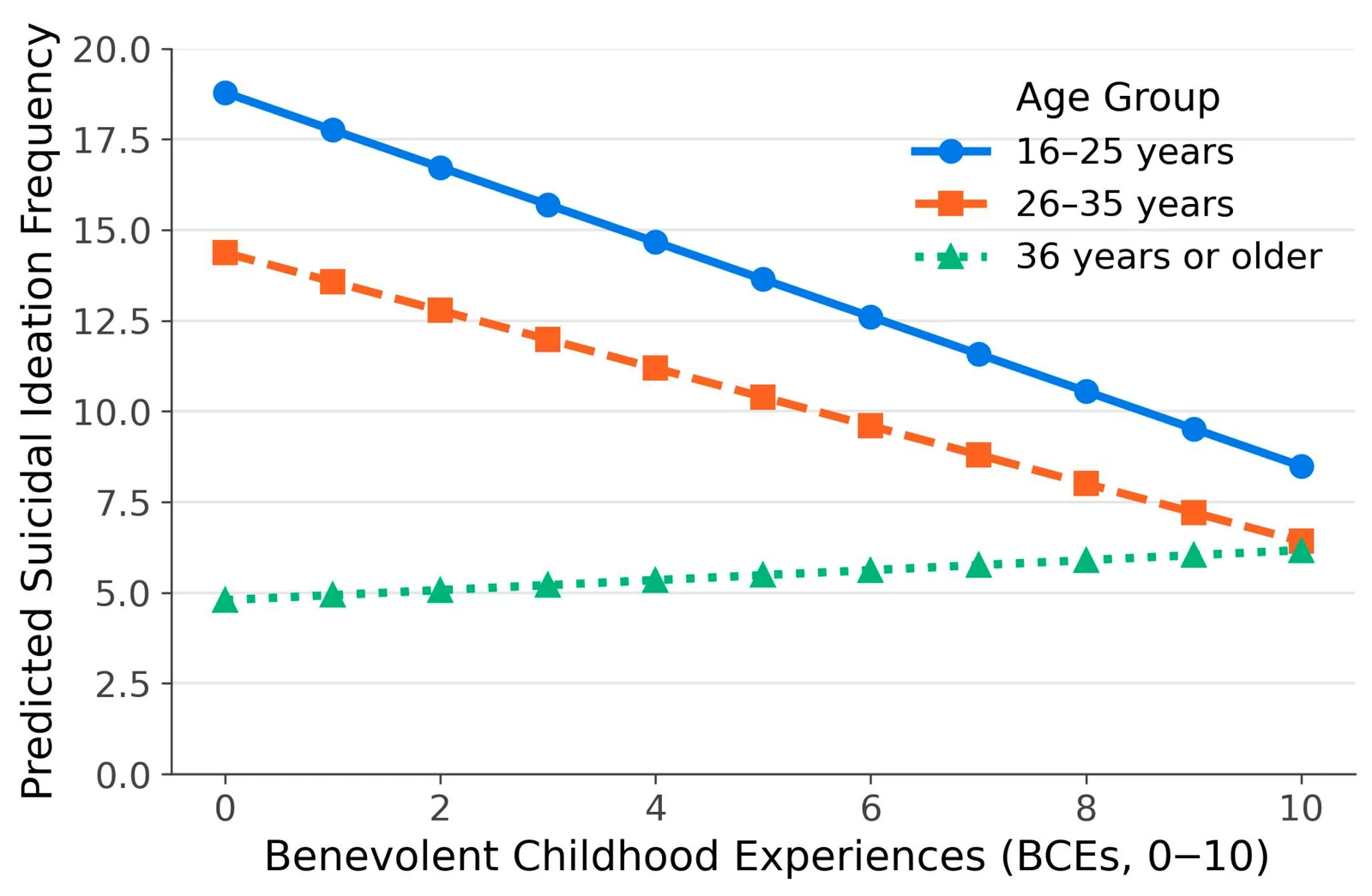
Predicted frequency of suicidal ideation as a function of benevolent childhood experiences by age group (categorical model, *N* = 451). Note. The figure was generated using the unstandardized coefficients from the model (Table 5), after removal of duplicate submissions. The slope of the 36 years or older group is based on 21 cases and is imprecisely estimated (95% CI [−0.702, 0.977]). The continuous-age model is displayed in Figures S1 and S2.

In the categorical model (Table 5, Figure 1), the interaction block added a small but significant change in *R*^2^ (Δ*R*^2^ = 0.013, *F* (2, 445) = 3.54, *p* = 0.030). This result was attributable entirely to the contrast between the 36 years or older group and the reference group (*b* = 1.167, 95% CI [0.292, 2.042], *p* = 0.009); the slope of the 26–35 years group did not differ from that of the 16–25 years group (*b* = 0.234, 95% CI [−0.388, 0.856], *p* = 0.460). The simple slopes show an inverse association among those aged 16–25 (*b* = −1.029, 95% CI [−1.277, −0.782]) and between ages 26 and 35 (*b* = −0.795, 95% CI [−1.366, −0.225]), whereas in the group aged 36 or older the slope was close to zero and imprecisely estimated (*b* = 0.138, 95% CI [−0.702, 0.977]), because this group comprises only 21 cases. The categorical evidence is therefore limited and should not be read as showing that the association “disappears after age 35”. To use all the information in the data, the model with age as a mean-centered continuous variable is reported in full in Table 6 (Figures S1 and S2). The BCEs × age interaction was positive (*b* = 0.061, 95% CI [0.026, 0.097], *p* < 0.001; HC3 95% CI [0.035, 0.088]; BCa bootstrap 95% CI [0.032, 0.087]; Δ*R*^2^ = 0.020, *F* (1, 447) = 11.48, *p* < 0.001), indicating a linear attenuation of the inverse association as age increases: the conditional slope of BCEs was −1.15 (95% CI [−1.41, −0.88]) at 18 years, −0.72 [−0.96, −0.48] at 25 years, −0.41 [−0.76, −0.06] at 30 years, and −0.11 [−0.61, 0.39] at 35 years. The Johnson–Neyman analysis located the upper bound of the region of significance at 30.7 years (bootstrap 95% CI for the age at which the slope reaches zero: 32.0–47.4 years); beyond that age, which concerns 39 participants (8.6%), the slope was not distinguishable from zero. The interaction was robust to the exclusion of influential observations (*b* = 0.076, *p* < 0.001; *n* = 438), of participants older than 50 years (*b* = 0.058, *p* = 0.003), to the log-transformation of the outcome (*b* = 0.0050, *p* = 0.005), and to MM-robust estimation (*b* = 0.063, *t* = 3.25); a quadratic age term did not improve the model (*p* = 0.112); a quadratic BCEs term was not significant in any model (all *p* > 0.30) and the RESET test did not indicate misspecification (*p* ≥ 0.18); and adjusting for university, field of study, residence area, and employment status left the interaction unchanged (*b* = 0.061, 95% CI [0.025, 0.096], *p* < 0.001; Table S9). With respect to multiple testing, the categorical interaction block (*p* = 0.030) does not meet the Bonferroni-adjusted threshold for the three moderation tests (α = 0.017; Holm-adjusted *p* = 0.090), whereas the continuous-age interaction does (Holm-adjusted *p* = 0.002). However, when the analysis was restricted to participants younger than 36 years (*n* = 430), the interaction was of similar size but no longer significant (*b* = 0.055, 95% CI [−0.006, 0.117], *p* = 0.078), which indicates that its statistical significance depends partly on the small number of older students. Taken together, these results provide partial support for H2: the inverse association is strongest in emerging adulthood and becomes weaker with age, but the age moderation should be regarded as exploratory rather than confirmatory and in need of replication in samples with a wider age distribution.

### 3.2. Model 2. Moderation by Gender

**Figure 2:**
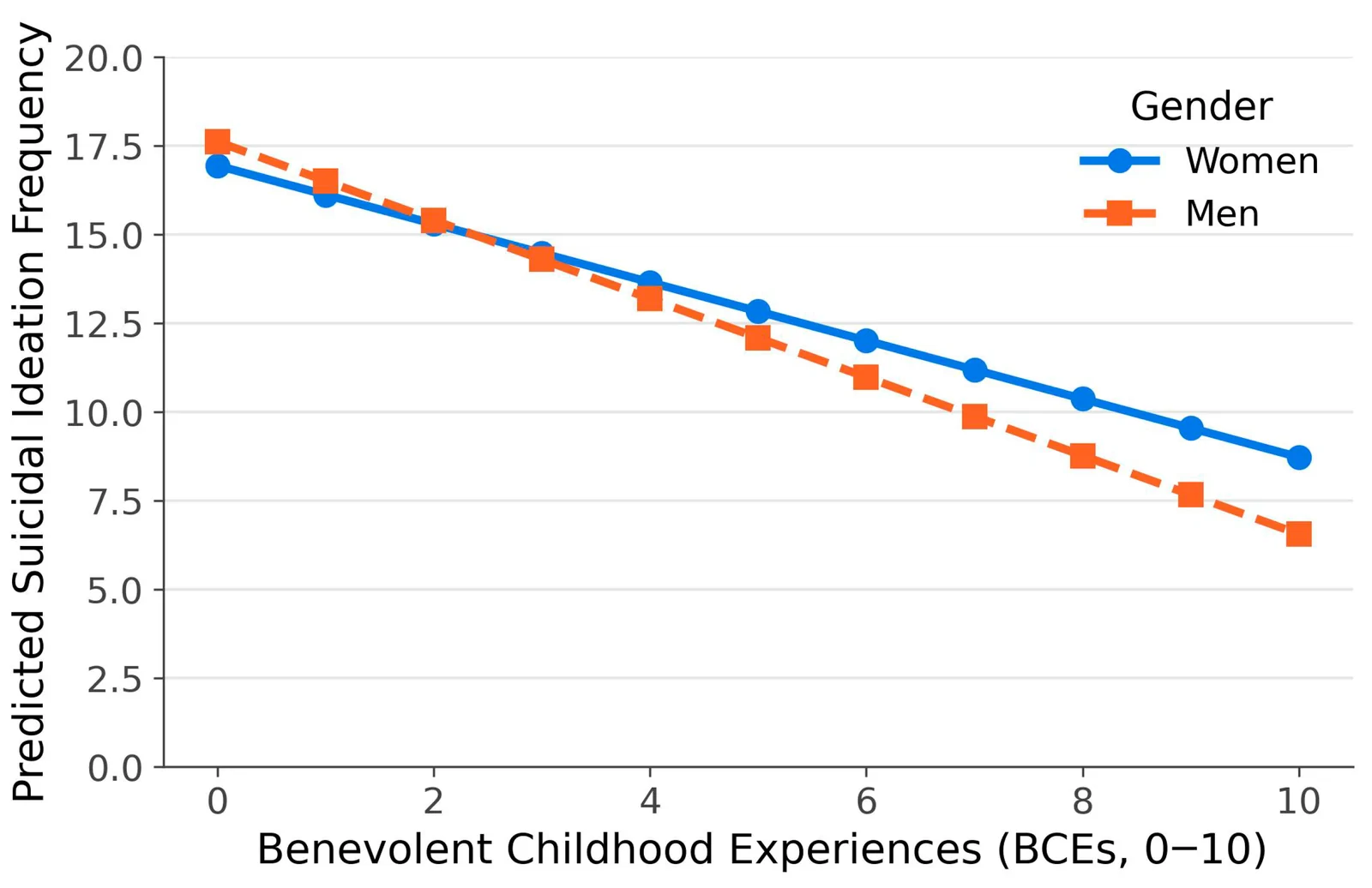
Predicted frequency of suicidal ideation as a function of benevolent childhood experiences by gender (*N* = 446). Note. The figure was generated using the model’s unstandardized coefficients (Table 7) after the removal of duplicate submissions.

In the second model, summarized in Table 7 and presented in Figure 2, the BCEs × gender interaction was not statistically significant, *b* = −0.284, 95% CI [−0.838, 0.269], *p* = 0.313 (Δ*R*^2^ = 0.002); therefore, H3 is not supported. The inverse association was present in both groups—women, *b* = −0.822, 95% CI [−1.080, −0.563]; men, *b* = −1.106, 95% CI [−1.596, −0.617]—and the difference between the two slopes was imprecisely estimated (95% CI from −0.84 to 0.27). This non-significant interaction does not demonstrate that the association is equivalent across genders: the study had 80% power to detect interactions of *f*^2^ ≥ 0.018 (Δ*R*^2^ ≈ 0.015), so smaller differences cannot be ruled out (Table S8); adjusting for available covariates left the interaction unchanged (*b* = −0.276, *p* = 0.323; Table S9). Women had a higher mean FIS score than men at the average level of BCEs (*b* = −1.577 for men, *p* = 0.003). The “prefer not to say” group (*n* = 5) was excluded from the inferential comparison due to insufficient sample size; its scores are reported in Table 2 for descriptive purposes only, and no inference regarding this group is drawn.

### 3.3. Model 3. Moderation by Marital Status

**Figure 3:**
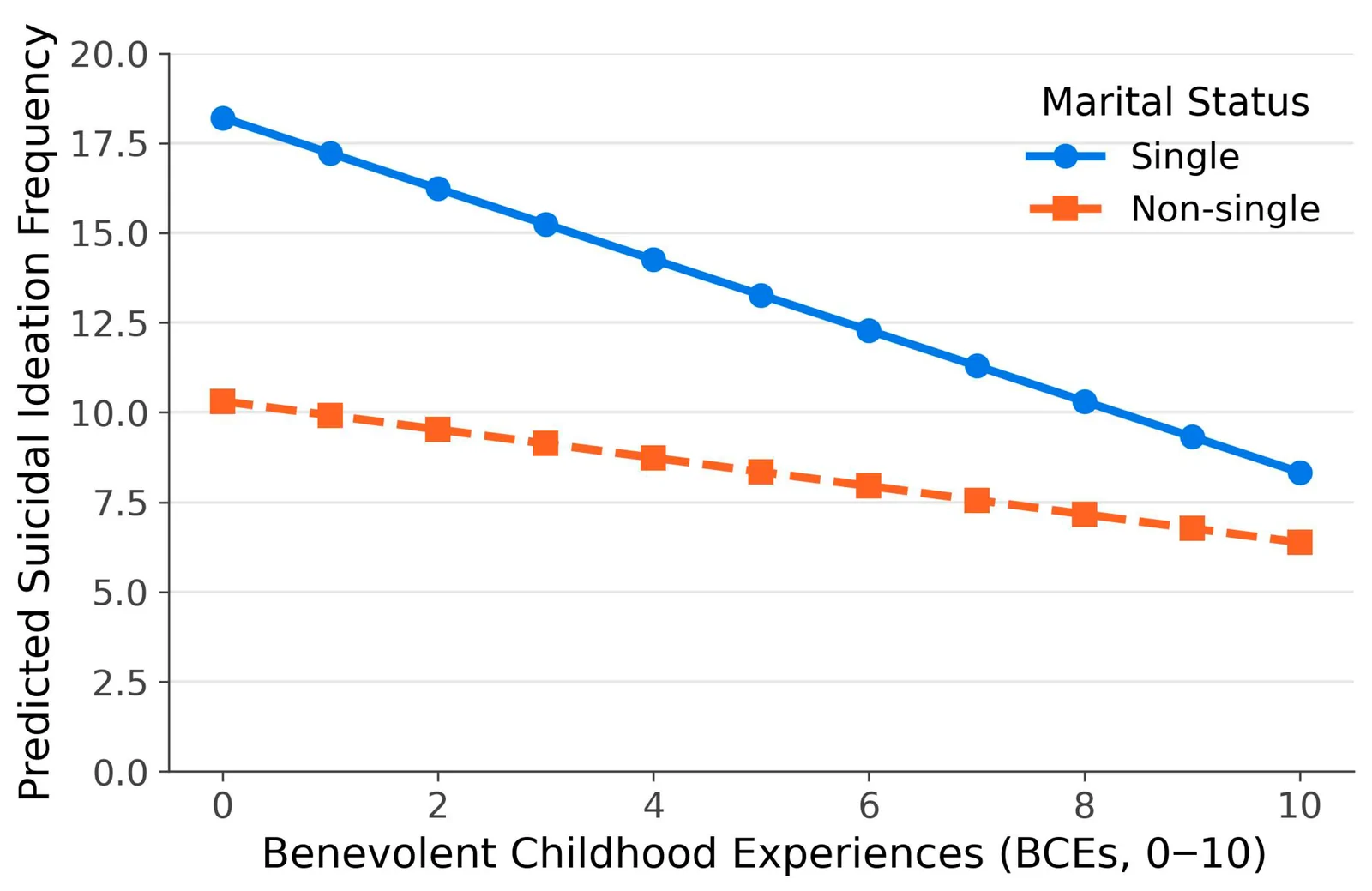
Predicted frequency of suicidal ideation as a function of benevolent childhood experiences by marital status (*N* = 451). Note. The figure was generated using the model’s unstandardized coefficients (Table 8) after the removal of duplicate submissions; the non-single line is based on 44 cases.

In the third model (Table 8), the interaction between BCEs and marital status was not statistically significant, *b* = 0.594, 95% CI [−0.113, 1.302], *p* = 0.100, with a small contribution to variance (Δ*R*^2^ = 0.005); therefore, H4 is not supported. The inverse association between benevolent childhood experiences and suicidal ideation was clearly present among single students (*b* = −0.988, 95% CI [−1.224, −0.751]), whereas among non-single students, the slope was smaller and imprecisely estimated (*b* = −0.393, 95% CI [−1.060, 0.273]), because this category is small (*n* = 44) and encompasses heterogeneous situations (cohabiting, married, divorced, and widowed). Given the width of the interval for the difference between slopes and the limited power of this test (observed *f*^2^ = 0.006 against a minimum detectable f^2^ of 0.018), the absence of a significant interaction should not be interpreted as evidence that the association is the same regardless of marital status (see Figure 3 and Table S8). Because the non-single category is conceptually heterogeneous, the model was re-estimated comparing partnered students (cohabiting or married, *n* = 41) with single students after excluding the three divorced or widowed participants: the interaction was similar (*b* = 0.556, 95% CI [−0.178, 1.290], *p* = 0.137), and the separate slopes for cohabiting (*b* = −0.67, *n* = 23) and married students (*b* = −0.19, *n* = 18) were both imprecisely estimated; adjusting for available covariates did not change the result (*b* = 0.533, *p* = 0.139; Table S9).

## 4. Discussion

The purpose of this study was to examine the relationship between benevolent childhood experiences and the frequency of suicidal ideation among Peruvian college students, considering age, gender, and marital status as potential sociodemographic moderators. The findings indicate an inverse association between benevolent childhood experiences and suicidal ideation that is consistent and statistically robust (*r* = −0.358, *p* < 0.001; robust to heteroscedasticity-consistent and bootstrap inference and to the exclusion of influential cases), although its modulation by sociodemographic variables is selective: only age showed evidence of moderation, and that evidence is exploratory. The regression models explain between 14% and 21% of the variance in suicidal ideation (R^2^ between 0.136 and 0.213). These percentages, while not accounting for the full variability of the phenomenon, are relevant given that these are moderation models involving complex psychosocial constructs assessed in non-clinical populations [37,38].

Regarding the main hypothesis, benevolent childhood experiences were significantly and negatively associated with the frequency of suicidal ideation. This result is consistent with the literature on early psychosocial capital and suicide risk, although, given the cross-sectional design and the absence of adjustment for confounders such as depression, current stress, socioeconomic circumstances, adverse childhood experiences, or psychiatric history, it documents an association and not a protective effect. A recent meta-analysis documented that benevolent childhood experiences are associated with fewer mental health symptoms in adulthood [12]. Narayan et al. [11] found that benevolent childhood experiences predict lower levels of psychopathology even among individuals with a history of early adversity, suggesting that their association with better outcomes is not limited to the absence of risk but rather reflects the active presence of resources that strengthen the individual’s regulatory capacity. The correlation coefficient obtained in the present study (*r* = −0.358) is of moderate magnitude and is consistent with previous evidence on positive experiences and suicidal ideation among young people [16,33], which provides external consistency.

These results can be interpreted in light of existing theory, bearing in mind that the mechanisms discussed below were not measured in the present study and are therefore proposed as plausible explanations supported by prior literature. Joiner’s interpersonal-psychological model of suicide [6] identifies frustrated belonging and the perception of being a burden as the central psychological states underlying this behavior. Positive life events associated with the presence of secure caregivers (87.1%), stable friendships (87.1%), and the opportunity to engage in enjoyable activities (90.7%) have been proposed in prior research as potential counterweights to both states [11,14], which offers a plausible explanation—albeit an untested one here—for the inverse association observed. Turecki et al. [2] noted that early relational protective factors reduce suicide risk through mechanisms of affect regulation and meaning making, which are consolidated during sensitive periods of development and are difficult to reverse in the face of subsequent adversity. One finding that deserves special attention is that about three-quarters of the participants in the present study (76.1%) did not endorse all ten experiences, while 23.9% endorsed all of them. This distribution suggests that a considerable proportion of college students enter higher education with limited protective childhood capital; on the basis of prior evidence [11,14], this could plausibly be associated with greater vulnerability to the academic and existential stressors characteristic of this stage of life, although the cross-sectional design does not allow this to be verified.

The item-by-item analysis provides further insight into this picture. The experiences with the lowest frequency of affirmative responses were those linked to autonomy and self-knowledge: item 9 (self-acceptance, 68.3%) and item 10 (predictable daily routine, 66.3%) recorded the lowest affirmation rates, while items related to external social connections exceeded 87%. In the sample studied, protective experiences of the relational-external type are more accessible than those associated with positive self-concept and internal household stability. This has direct implications for the design of interventions: it is not enough to promote social integration on campus if, at the same time, the intrapsychic resources that enable students to translate these connections into schemas of self-worth are not addressed [7,9]. On the FSII, the item with the highest mean score was the one referring to the question of what would happen if one were to end one’s own life (*M* = 2.24); however, item-level means cannot by themselves characterize the reflective or impulsive quality of suicidal thinking, which would require dedicated measures of rumination or impulsivity [59,60] and is not examined here.

Regarding moderators, age was the only variable for which evidence of moderation was obtained. The categorical model yielded a small but significant interaction block (Δ*R*^2^ = 0.013, *p* = 0.030) that rested entirely on 21 students aged 36 years or older, whereas the continuous-age model—which does not depend on arbitrary cut-offs—showed a linear attenuation of the inverse association with age (*b* = 0.061, 95% CI [0.026, 0.097]; Δ*R*^2^ = 0.020) that was robust to influential observations, to the exclusion of participants older than 50 years, to the transformation of the outcome, and to robust estimation, but that was no longer significant when the analysis was restricted to students younger than 36 years. The direction of the finding is theoretically consistent: the inverse association between BCEs and suicidal ideation is strongest in the 16–25 age group, which accounts for 82.3% of the sample, weakens progressively with age, and is no longer distinguishable from zero beyond approximately 31 years. Because the older part of the age distribution is sparsely represented, and because the categorical interaction does not survive a Bonferroni correction for the three moderation tests (whereas the continuous-age interaction does), we regard this result as exploratory rather than confirmatory and as partial support for H2. This progression is consistent with Arnett [27], who explains that emerging adulthood is a period of maximum receptivity to resources internalized during childhood, insofar as the tasks of identity exploration and the consolidation of emotional bonds characteristic of this stage would reactivate the internal working models formed within the early family unit.

A complementary, untested explanation is that the closer temporal proximity of emerging adults to their childhood experiences, together with the developmental processes of stress regulation that are still consolidating at that age [9,61], makes the association more visible in the younger group; because neither affective regulation nor any neurobiological indicator was measured in this study, this explanation is offered only as a hypothesis for future research. In Peru, college students in their first years of study report higher levels of suicidal ideation, especially during the first semesters of academic adjustment [24,62], making the age-related attenuation of this association a finding with potential preventive relevance.

Unlike age, gender did not statistically significantly moderate the relationship between BCEs and suicidal ideation (*b* = −0.284, 95% CI [−0.838, 0.269], *p* = 0.313, Δ*R*^2^ = 0.002), and the inverse association was present among both women and men. This finding should be interpreted cautiously: it indicates that no significant difference between the slopes was detected, not that the slopes are equivalent, because the study was powered to detect only interactions of *f*^2^ ≥ 0.018 and the confidence interval for the slope difference is wide. Within that limit, the data provide no evidence that gender-differentiated socialization alters the strength of this association. Eagly and Wood’s social roles theory [63] would suggest that women, who are socialized to express emotions and actively seek interpersonal support, would be better able to capitalize on relational resources such as bonds with friends or the presence of supportive adults. One plausible explanation, consistent with prior literature but not directly tested here, is that the mechanisms through which BCEs relate to lower suicidal ideation are largely shared by women and men [9,18,19]; this interpretation should be regarded as a hypothesis to be examined in adequately powered studies that measure such mechanisms directly.

The third moderator showed a similar pattern: marital status was not a significant moderator of the relationship between protective childhood experiences and suicidal ideation (*b* = 0.594, 95% CI [−0.113, 1.302], *p* = 0.100, Δ*R*^2^ = 0.005). The inverse association was clear among single students, whereas among the 44 non-single students it was smaller and imprecisely estimated (*b* = −0.393, 95% CI [−1.060, 0.273]); given the limited power of this test, the result does not establish that the association holds regardless of relationship status. Joiner’s interpersonal theory [6] and Cutrona and Russell’s social support compensation model [36] suggest that psychosocial resources internalized during childhood act as functional substitutes for adult relational support. The absence of a significant interaction is compatible with the possibility that the association between benevolent childhood experiences and suicidal ideation does not depend on the availability of partner support in adulthood [6,36], although the present data cannot establish this, and the mechanisms involved were not measured.

Prior literature has proposed intrapersonal mechanisms—adaptive emotion-regulation strategies, a positive self-concept, and mentalization skills—through which early protective experiences might relate to lower suicidal ideation irrespective of current relational conditions [9,11,14,18,64]; these mechanisms were not assessed here and remain hypotheses for future research.

On the instrumental level, the Benevolent Childhood Experiences Scale yielded a KR-20 = 0.66, a marginal value that is nevertheless expected given the heterogeneous nature of the construct: BCEs constitute, by design, an index of the accumulation of qualitatively diverse experiences [14]. The mean inter-item correlation value ($\bar{r}$ij = 0.161) falls within the optimal range for broadly defined multidimensional constructs [49]. The corrected item-total correlations for the BCEs ranged from 0.181 to 0.414, with several items in the weak or borderline range, which reflects the breadth of the protective domain that the scale aims to capture but also limits the precision of the total score. The confirmatory and Rasch analyses added during revision (Table 4; Supplementary Section S1) support an acceptable unidimensional structure (WLSMV CFA: CFI = 0.955, RMSEA = 0.038) with moderate loadings (0.32–0.75) and a categorical omega of 0.82, an ordered set of item difficulties, and acceptable item fit, although the Andersen test suggested some lack of parameter invariance across score levels and person-separation reliability was low. Because the Spanish version has not been validated in Peru and no external criterion scale was administered, these results should be read as preliminary evidence of adequacy for research use, not as a validation study.

For its part, the Suicidal Ideation Frequency Inventory presented a homogeneous psychometric profile: α = 0.94, item-total correlations between 0.810 and 0.872, and a mean inter-item correlation of 0.756, above the optimal range and consistent with a narrow unitary construct. The categorical CFAs confirmed this structure, with loadings of 0.90–0.94, categorical omega = 0.96, and AVE = 0.83 [47], and the two-factor model with BCEs fitted significantly better than a single common factor, with a latent correlation of −0.53; both measures are therefore related but empirically distinguishable, a necessary condition for the interpretation of the moderation models. These indicators are comparable to those reported in validation studies of suicide ideation scales among Latin American university students [24,26].

In interpreting these findings, statistical significance should be distinguished from clinical significance and from preventive relevance. The association between BCEs and suicidal ideation is statistically robust and of moderate size (*r* = −0.36; each additional benevolent experience corresponds to about 0.9 fewer points on the 5–25 FIS scale, i.e., 0.18 SD), and it extends the cross-cultural scope of the benevolent experiences model to a Latin American university sample [10,14]; however, its clinical significance cannot be established in the absence of validated thresholds for the FSII or of clinical outcomes, and its preventive relevance is a hypothesis that only longitudinal and intervention studies can test. The age moderation is statistically detectable in the continuous model but small (Δ*R*^2^ = 0.020), does not survive correction for multiple testing in its categorical form, and is therefore exploratory; and the non-significant gender and marital-status interactions are uninformative about equivalence across groups.

Regarding the magnitude of the phenomenon, the tertile groups of Table 2 are sample-specific by construction and cannot be read as prevalence estimates of high suicide risk; the only item-based descriptive indicator is that 23.1% of participants (104 of 451) answered “sometimes”, “often”, or “almost every day” to the item “how often have you thought about killing yourself?” (item 4 of the FSII) in the past 12 months, a figure that warrants attention but that is not equivalent to a clinical assessment of risk. This descriptive finding underlines the relevance of mental health support in university settings [3,4,26]; however, it does not justify the incorporation of BCEs screening into admission protocols, a recommendation that would be premature on the basis of a cross-sectional convenience sample and that could raise ethical concerns about labeling and stigmatization of students on the basis of their childhood histories. The inclusion of the brief BCEs scale, which showed acceptable psychometric indicators in the present sample, might, in future research, help to characterize the early protective resources of students who seek psychological support services [9,27,34,65,66,67]; however, given the convenience sampling, the lack of a published Peruvian validation of the scale, and the ethical considerations noted above, no screening application is recommended at this stage.

### Limitations

The main limitation is the cross-sectional design, which prevents the establishment of causal relationships between BCEs and suicidal ideation. Although life-course theory posits that childhood experiences temporally precede mental health outcomes in adulthood, this causal sequence cannot be verified with the data from the present study [11,13]. The study did not measure depression, current stress, socioeconomic circumstances, adverse childhood experiences, or psychiatric history, so the association between BCEs and suicidal ideation is unadjusted for these potential confounders and cannot be interpreted as a protective effect. Added to this are the non-probability convenience sampling and the sample composition—with a predominance of psychology students (69.0%) and women (74.9%)—which limit the generalizability of the findings; in addition, 28 participants had submitted the questionnaire more than once, which was detected through the e-mail addresses collected and corrected by retaining a single submission per participant. The measures were self-reported, and recall of childhood experiences may be biased by current emotional state. The imbalance in age groups and marital status reduces the precision of slopes in the minority categories, and the moderation tests had 80% power only for interaction effects of *f*^2^ ≥ 0.018–0.021, so the non-significant gender and marital-status interactions do not establish equivalence, and the age moderation, which depends partly on 21 students aged 36 years or older, is exploratory. The FIS score is bounded, positively skewed, and shows a floor effect; heteroscedasticity was detected in all models and was addressed with HC3 and bootstrap inference, whose conclusions coincided with those of ordinary least squares. No a priori power analysis was conducted, and no adjustment for multiple testing was prespecified: the categorical age interaction does not survive a Bonferroni correction for the three moderation tests, which is a further reason to regard the age moderation as exploratory. The dichotomization of marital status combined cohabiting, married, divorced, and widowed students, situations that differ socially; the sensitivity analysis restricted to partnered versus single students yielded a similar estimate, but this variable should be treated as a coarse proxy of relational status. Adjustment for the covariates available in the dataset (university, field of study, residence area, employment) did not alter the results, but these are not the confounders of greatest concern. The Spanish version of the BCEs scale has not yet undergone published psychometric validation in the Peruvian population; its classical reliability in the present sample was marginal (KR-20 = 0.66), its unidimensional fit was acceptable, and no external criterion scale was available to examine convergent validity. Finally, emotional regulation, cited as an explanatory mechanism, was not measured directly; therefore, it should be treated as a hypothesis to be tested in future studies. Consequently, the explanatory mechanisms discussed above—the hypothesized buffering role of BCEs, age-related affect-regulation processes, and the intrapsychic pathways invoked to account for the absence of gender and marital-status moderation—were not measured in this study and should be read as plausible explanations derived from prior literature rather than as findings of the present research.

## 5. Conclusions

The results of this study indicate that benevolent childhood experiences are inversely associated with suicidal ideation among Peruvian college students, with an association of moderate magnitude and a direction consistent with the postulates of the biopsychosocial model of resilience; because the design is cross-sectional and unadjusted for relevant confounders, this association does not demonstrate a protective effect. The inverse association weakened with age and was strongest in emerging adulthood, a result that is presented as exploratory, whereas no significant differences by gender or marital status were detected, which, given the limited power of these tests, should not be read as evidence that the association is independent of these variables. These findings have potential implications for suicide prevention in university settings, as they identify benevolent childhood experiences as a domain that merits further investigation in university mental health research. Nevertheless, given the non-probability convenience sample and the absence of a published psychometric validation of the Spanish version of the BCEs scale in the Peruvian population, any clinical or preventive use of the scale would be premature at this stage. The confirmatory and Rasch analyses reported here support the use of the scale for research purposes, notwithstanding its marginal classical reliability (KR-20 = 0.66), whereas clinical–preventive applications in the Peruvian and Latin American contexts should await formal validation studies, and screening on the basis of childhood histories raises ethical concerns that go beyond psychometrics. Continuing this line of research using longitudinal designs and more diverse and balanced samples is not only methodologically important but also ethically necessary, given the frequency of suicidal ideation documented in the university population studied.

## Figures and Tables

**Table 1:** Sociodemographic characteristics of the participants (*N* = 451).

Variable	Category	*n*	%
Age	16–25 years	371	82.3
	26–35 years	59	13.1
	36 years or older	21	4.7
	*M = 22.12 SD = 6.23 Range: 17–66*		
Gender	Female	338	74.9
	Male	108	23.9
	Would rather not say	5	1.1
Marital Status	Single	407	90.2
	Living with a partner	23	5.1
	Married	18	4.0
	Widowed	2	0.4
	Divorced	1	0.2
Employment	Not working	272	60.3
	Temporary employment	74	16.4
	Retail clerk	39	8.6
	Self-employed	37	8.2
	Full-time job	29	6.4
College Level	Cycles I–IV (early years)	297	66.9
	Cycles V–VII (intermediate)	51	11.5
	Cycles VIII–X (final years)	96	21.6
	*M = 5.01, SD = 2.58 (n = 444)*		
Residence Area	Urban	365	80.9
	Rural	86	19.1
Professional Career	Psychology	311	69.0
	Business administration	72	16.0
	Human medicine	14	3.1
	Early childhood education	11	2.4
	Accounting	8	1.8
	Civil engineering	7	1.6
	Nursing	7	1.6
	Other majors	21	4.7

Note. *M* = mean; *SD* = standard deviation. Percentages for the university cycle were calculated based on *n* = 444 cases (7 missing). Age categories (16–25, 26–35, and ≥36 years) were prespecified on theoretical grounds (see Section 2.4) and are labeled by their age ranges. Business administration combines the responses “Administración” and “Administración de Empresas”, which designate the same program. Other majors (*n* = 21) included law (*n* = 5), systems engineering (*n* = 3), obstetrics (*n* = 2), environmental engineering (*n* = 2), and nine programs with one student each (industrial, mechatronic, chemical, and business management engineering; communication sciences; primary education; education, unspecified; humanities; and one unspecified program).

**Table 2:** Descriptive distribution of the benevolent childhood experiences (BCEs) and frequency of suicidal ideation (FIS) scores by gender (sample-based tertile groups, for descriptive purposes only).

(**a**) Benevolent childhood experiences (BCEs): dichotomous scale, with a range of 0–10.					
**Level**	**Range**	**Total *n* (%)**	**Women *n* (%)**	**Men *n* (%)**	**Prefer Not to Say *n* (%)**
Lower tertile	0–7	157 (34.8)	120 (35.5)	33 (30.6)	4 (80.0)
Middle tertile	8–9	186 (41.2)	138 (40.8)	47 (43.5)	1 (20.0)
Upper tertile	10	108 (23.9)	80 (23.7)	28 (25.9)	0 (0.0)
M (SD)	—	7.92 (1.96)	7.91 (1.97)	8.06 (1.84)	5.60 (2.41)
(**b**) Frequency of suicidal ideation (FIS; FSII total score): 1–5 Likert scale, with a range of 5–25.					
**Level**	**Range**	**Total *n* (%)**	**Women *n* (%)**	**Men *n* (%)**	**Prefer Not to Say *n* (%)**
Lower tertile	5–6	170 (37.7)	116 (34.3)	54 (50.0)	0 (0.0)
Middle tertile	7–12	134 (29.7)	100 (29.6)	32 (29.6)	2 (40.0)
Upper tertile	13–25	147 (32.6)	122 (36.1)	22 (20.4)	3 (60.0)
M (SD)	—	10.09 (5.13)	10.44 (5.17)	8.71 (4.63)	15.60 (6.35)
(**c**) Frequency of affirmative responses by item—BCEs.					
**Item**	**Description**			***n*** **(Yes = 1)**	**% (Yes)**
1	Safe caregiver			393	87.1
2	Good friend			393	87.1
3	Comforting beliefs			355	78.7
4	I liked school			410	90.9
5	A teacher who cared			364	80.7
6	Good neighbors			316	70.1
7	External support adult			326	72.3
8	Opportunities to have fun			409	90.7
9	Self-satisfaction			308	68.3
10	Predictable daily routine			299	66.3
(**d**) Descriptive statistics by item—FIS.					
**Item**	**Description**			**M**	**SD**
1	Intended to cause harm			1.96	1.08
2	Believed they did not deserve to live			2.03	1.16
3	Asked what would happen if they ended their life			2.24	1.23
4	Thought about committing suicide			1.75	1.01
5	Wished not to exist			2.11	1.24

Note. The tertile groups are purely descriptive, where cut-off points correspond to the empirical 33rd and 67th percentiles of the present sample (*N* = 451) and are therefore sample-specific by construction; they are not clinical thresholds, and the proportion of cases in each group must not be read as a prevalence estimate. No validated clinical cut-off is available for the FSII, so all inferential analyses treat the FIS score as a continuous variable. BCEs: lower tertile = 0–7, middle = 8–9, and upper = 10; range 0–10 (10 dichotomous items; 1 = presence of experience). FIS: lower tertile = 5–6, middle = 7–12, and upper = 13–25; range 5–25 (5 Likert-scale items, 1–5). Gender was selected as a contrast variable due to its theoretical basis in suicidal ideation: women have higher mean ideation scores (10.44 vs. 8.71), while men exhibit a higher risk of completed attempts [5,48]. The “prefer not to say” group (*n* = 5) is reported for descriptive purposes only; its sample size does not allow for inferences. Cell entries in panels (a) and (b) are *n* (%), except for the last rows, which report M (SD). Group sizes: total *N* = 451; women *n* = 338; men *n* = 108; and prefer not to say *n* = 5.

**Table 3:** Classical psychometric indicators of BCEs and FSII: reliability, item discrimination, and homogeneity.

(**a**) Reliability.															
**Scale/Dimension**				**Number of Items**			***N***		**Coefficient**					**Value**	**Interpretation**
BCEs—Benevolent Childhood Experiences				10			451		KR-20					0.66	Marginal
FSII—Suicidal Ideation Frequency Inventory				5			451		Cronbach’s α					0.94	Excellent
(**b**) Corrected item-total correlations (ritc).															
**Scale**	**Item**	**Abbreviated Description**									**ritc**		**Discrimination**		
BCEs	1	Safe caregiver									0.181		Weak		
	2	Good friend									0.371		Acceptable		
	3	Comforting beliefs									0.217		Borderline		
	4	I liked school									0.268		Borderline		
	5	A teacher who cared									0.300		Acceptable		
	6	Good neighbors									0.414		Good		
	7	External support adult									0.323		Acceptable		
	8	Opportunities to have fun									0.388		Acceptable		
	9	Self-satisfaction									0.393		Acceptable		
	10	Predictable daily routine									0.358		Acceptable		
FSII	1	Intent on hurting himself									0.825		Good		
	2	Did not deserve to live									0.872		Good		
	3	End their life									0.821		Good		
	4	Thought about committing suicide									0.810		Good		
	5	Wished not to exist									0.857		Good		
(**c**) Homogeneity—mean inter-item correlation ($\bar{r}$ij).															
**Scale**	$\bar{r}$ **ij**		**Optimal Range** [49]					**Assessment**							
BCEs	0.161		0.15–0.50					Acceptable (expected multidimensionality)							
FSII	0.756		0.15–0.50					Above the range (unitary construct)							
(**d**) Evidence of construct validity.															
**Type of Evidence**					**Indicator**	**Value**				**Direction**		**Interpretation**			
Relationship with other variables (external criterion)					r (BCEs × FIS)	−0.358				Negative		Consistent with theory: more BCEs → less FIS			
Internal structure of BCEs					ritc min–max	0.18–0.41				Positive		Items consistent with the general construct			
Internal structure of FSII					ritc min–max	0.81–0.87				Positive		High internal cohesion; unitary construct			

Note. KR-20 = Kuder–Richardson formula 20 (reliability for dichotomous items). α = Cronbach’s alpha (polytomous items). ritc = corrected item-total correlation (the item score is subtracted from the total): ≥0.40 = good, 0.30–0.39 = acceptable, 0.20–0.29 = borderline, <0.20 = weak [44]. $\bar{r}$ij = mean inter-item correlation; optimal range 0.15–0.50 [49]. The negative correlation between BCEs and FIS (*r* = −0.358, *p* < 0.001) provides evidence of validity based on relationships with other variables: benevolent childhood experiences are inversely associated with suicidal ideation, in the direction predicted by the Resilience and Protective Factors model [50,51]. *N* = 451. Confirmatory (CFA) and Rasch-based evidence is reported in Table 4 and in the Supplementary Materials (Section S1).

**Table 4:** Confirmatory factor analyses (WLSMV estimator on polychoric/tetrachoric correlations) of BCEs and FSII: fit indices, standardized loadings, and model-based reliability.

(**a**) Fit indices of the measurement models (N = 451).										
**Model**		**Scaled χ^2^ (*df*), *p***	**CFI**	**TLI**		**RMSEA [90% CI]**		**SRMR**		**Interpretation**
BCEs—one factor (10 items)		58.04 (35), 0.009	0.955	0.942		0.038 [0.019, 0.055]		0.082		Accept^a^ble fit a
FSII—one factor (5 items)		33.94 (5), <0.001	0.998	0.996		0.113 [0.079, 0.151]		0.013		Good fit; RMSEA inflated by *df* = 5
Two factors—BCEs + FSII (15 items)		177.77 (89), <0.001	0.993	0.992		0.047 [0.037, 0.057]		0.084		Good fit; *r*(BCEs, FIS) = −0.53 [−0.62, −0.44]
One factor—all 15 items		473.85 (90), <0.001	0.971	0.967		0.097 [0.089, 0.106]		0.143		Worse than two factors: Δχ^2^(1) = 82.90, *p* < 0.001
(**b**) Standardized loadings and model-based reliability of the one-factor models.										
**Construct**	**Standardized Loadings (Range)**				**ω (Categorical)**		**AVE**		**Classical Reliability**	
BCEs (10 dichotomous items)	0.32–0.75 (all *p* < 0.001)				0.82		0.33		KR-20 = 0.66; ordinal α = 0.82	
FSII (5 Likert items)	0.90–0.94 (all *p* < 0.001)				0.96		0.83		α = 0.94; ordinal α = 0.96	

Note. Estimator: WLSMV (diagonally weighted least squares with mean- and variance-adjusted test statistic) on the tetrachoric (BCEs) and polychoric (FSII) correlation matrices; scaled fit indices are reported. CFI = comparative fit index; TLI = Tucker–Lewis index; RMSEA = root mean square error of approximation; SRMR = standardized root mean square residual; ω = model-based reliability computed from the standardized loadings of the categorical CFA (categorical omega); AVE = average variance extracted from the same loadings. Δ*χ*^2^ = S^a^torra–Bentler scaled chi-square difference test between the two-factor and the single-factor models. a The BCEs are conceived as a cumulative index of heterogeneous experiences; the acceptable one-factor fit, the moderate loadings, and the low AVE are consistent with that conception, and the AVE ≥ 0.50 convergent-validity criterion is not applied to this index. Full item-level results, the Rasch analysis, and the known-groups comparisons are reported in the Supplementary Materials (Tables S1–S4).

**Table 5:** Summary of the categorical age moderation model (BCEs mean-centered).

Predictor	*B*	*SE*	*t*	*p*	95% CI
Benevolent childhood experiences	−1.029	0.126	−8.181	<0.001	[−1.277, −0.782]
Age: 26–35 years (vs. 16–25)	−2.561	0.650	−3.939	<0.001	[−3.839, −1.283]
Age: 36 or older (vs. 16–25)	−4.741	1.037	−4.571	<0.001	[−6.780, −2.703]
BCEs × 26–35 years interaction	0.234	0.316	0.739	=0.460	[−0.388, 0.856]
BCEs × 36 years or older interaction	1.167	0.445	2.622	=0.009	[0.292, 2.042]

Note. *N* = 451 (16–25 years: 371; 26–35 years: 59; 36 years or older: 21). BCEs were mean-centered (*M* = 7.92). *R*^2^ = 0.200, *F* (5, 445) = 22.19, *p* < 0.001. Reference group: 16–25 years. The change in R^2^ attributable to the interaction block was 0.013, *F* (2, 445) = 3.54, *p* = 0.030. Simple slopes of the BCEs with 95% CIs: 16–25 years, *b* = −1.029 [−1.277, −0.782]; 26–35 years, *b* = −0.795 [−1.366, −0.225]; 36 years or older, *b* = 0.138 [−0.702, 0.977]. HC3 and BCa bootstrap 95% CIs for the BCEs × 36 years or older term: [0.821, 1.513] and [0.846, 1.627]; the interaction block remained significant after excluding influential cases (*p* = 0.023) and in MM-robust regression, and was borderline with a log-transformed outcome (*p* = 0.051) (Tables S6 and S7).

**Table 6:** Continuous-age moderation model (BCEs and age mean-centered).

Predictor	*B*	*SE*	*t*	*p*	95% CI
Benevolent childhood experiences (centered)	−0.896	0.111	−8.105	<0.001	[−1.113, −0.679]
Age in years (centered)	−0.234	0.035	−6.595	<0.001	[−0.303, −0.164]
BCEs × age interaction	0.061	0.018	3.389	<0.001	[0.026, 0.097]

Note. *N* = 451. *R*^2^ = 0.213, *F* (3, 447) = 40.30, *p* < 0.001; ΔR^2^ for the interaction = 0.020, *F* (1, 447) = 11.48, *p* < 0.001. Means used for centering: BCEs = 7.92; age = 22.12 years. HC3 robust 95% CI for the interaction: [0.035, 0.088]; BCa bootstrap 95% CI (5000 resamples): [0.032, 0.087]. Johnson–Neyman upper bound of the region of significance: 30.7 years. Conditional slopes of BCEs at selected ages, sensitivity analyses, and diagnostics are reported in Tables S5–S8 and Figures S1–S3.

**Table 7:** Summary of the gender moderation model (BCEs mean-centered).

Predictor	*B*	*SE*	*t*	*p*	95% CI
Benevolent childhood experiences	−0.822	0.131	−6.250	<0.001	[−1.080, −0.563]
Gender (male vs. female)	−1.577	0.526	−2.998	=0.003	[−2.611, −0.543]
BCEs × gender interaction	−0.284	0.282	−1.010	=0.313	[−0.838, 0.269]

Note. *N* = 446 (women: 338; men: 108; the “prefer not to say” group, *n* = 5, was excluded). Coding: 0 = female, 1 = male; BCEs were mean-centered. *R*^2^ = 0.136, *F* (3, 442) = 23.22, *p* < 0.001. The change in R^2^ attributable to the interaction was 0.002, *F* (1, 442) = 1.02, *p* = 0.313. Simple slopes with 95% CIs: women, *b* = −0.822 [−1.080, −0.563]; men, *b* = −1.106 [−1.596, −0.617]. HC3 and BCa bootstrap 95% CIs for the interaction: [−0.905, 0.336] and [−0.876, 0.353]. Sensitivity of the interaction test: minimum detectable *f*^2^ = 0.018 (Δ*R*^2^ ≈ 0.015) with 80% power; observed *f*^2^ = 0.002.

**Table 8:** Summary of the marital status moderation model (BCEs mean-centered).

Predictor	*B*	*SE*	*t*	*p*	95% CI
Benevolent childhood experiences	−0.988	0.121	−8.192	<0.001	[−1.224, −0.751]
Marital status (non-single vs. single)	−3.190	0.752	−4.241	<0.001	[−4.668, −1.712]
BCEs × marital status interaction	0.594	0.360	1.651	=0.100	[−0.113, 1.302]

Note. *N* = 451 (single: 407; non-single: 44). Coding: 0 = single, 1 = not single; BCEs were mean-centered. *R*^2^ = 0.165, *F* (3, 447) = 29.40, *p* < 0.001. The change in R^2^ attributable to the interaction was 0.005, *F* (1, 447) = 2.73, *p* = 0.100. Simple slopes with 95% CIs: single, *b* = −0.988 [−1.224, −0.751]; non-single, *b* = −0.393 [−1.060, 0.273]. HC3 and BCa bootstrap 95% CIs for the interaction: [−0.112, 1.300] and [−0.127, 1.125]. Sensitivity of the interaction test: minimum detectable *f*^2^ = 0.018 (Δ*R*^2^ ≈ 0.015) with 80% power; observed *f*^2^ = 0.006. Adjusting for age, which is strongly associated with marital status, did not change the conclusion (interaction *b* = 0.618, *p* = 0.080).

## Data Availability

The de-identified dataset supporting the conclusions of this article (with e-mail addresses removed) and the analysis code (SPSS/PROCESS syntax and the R scripts used for the confirmatory factor, Rasch, diagnostic, robust, and bootstrap analyses) are available from the corresponding author upon reasonable request, subject to the conditions of the ethics approval.

## Supplementary Materials

The following supporting information can be downloaded at: https://www.mdpi.com/article/10.3390/healthcare14213574/s1, Section S1. Psychometric analysis of the Spanish BCEs scale and the FSII: item endorsement and tetrachoric correlations (Table S1), confirmatory factor analyses (Table S2), Rasch model (Table S3), and known-groups and convergent evidence (Table S4); Section S2. Regression diagnostics (Table S5; Figure S3); Section S3. HC3 and bootstrap confidence intervals (Table S6) and sensitivity analyses (Table S7); Section S4. Continuous-age model, Johnson–Neyman analysis (Figures S1 and S2), simple slopes, and sensitivity of the interaction tests (Table S8); Section S5. Additional sensitivity analyses (covariate-adjusted models, alternative coding of marital status, linearity checks, and multiplicity-adjusted *p*-values; Table S9).

## Abbreviations

The following abbreviations are used in this manuscript:

AVE	Average Variance Extracted
BCEs	Benevolent Childhood Experiences (construct and scale)
CI	Confidence Interval
CR	Composite Reliability (replaced by ω in the revised Table 4)
FIS	Frequency of Suicidal Ideation (construct and score)
FSII	Suicidal Ideation Frequency Inventory
KR-20	Kuder–Richardson Formula 20
ritc	Corrected Item-Total Correlation
WHO	World Health Organization
CFA	Confirmatory Factor Analysis
HC3	Heteroscedasticity-Consistent Standard Errors (type 3)
WLSMV	Weighted Least Squares with Mean- and Variance-Adjusted Statistics
ω	McDonald’s Omega (model-based reliability)
