# Supplementary material for: Benevolent Childhood Experiences and Suicidal Ideation Among College Students: A Moderation Analysis by Age, Gender, and Marital Status
Source: Healthcare (Basel). 2026 Oct 22;14(21):3574. doi: 10.3390/healthcare14213574 (PMC13762759; doi:10.3390/healthcare14213574)
Supplement: Supplementary file 1 [file healthcare-14-03574-s001.zip › healthcare-4528112-supplementary.pdf]

Supplementary Materials

Benevolent Childhood Experiences and Suicidal Ideation Among College Students: A Moderation Analysis by Age, Gender, and Marital Status

Manuscript healthcare-4528112. All analyses were performed on the deduplicated analytical sample (N = 451; one submission per participant) with R 4.3.3 (packages lavaan 0.6, eRm, psych, sandwich, lmtest, car, MASS, boot). Sections S1–S5 correspond to the analyses requested during peer review. Unless stated otherwise, BCEs = total score of the Benevolent Childhood Experiences Scale (0–10); FIS = total score of the Suicidal Ideation Frequency Inventory (FSII; 5–25).

Section S1. Psychometric analysis of the Spanish BCEs scale and of the FSII

S1.1. Item endorsement and tetrachoric correlations

Table S1 reports the proportion of affirmative responses to each BCEs item and the tetrachoric correlation matrix on which the confirmatory factor analysis was estimated. Sampling adequacy was acceptable (Kaiser–Meyer–Olkin = 0.75; Bartlett's test $\chi^2(45) = 1394.23$, $p < .001$). The first eigenvalue of the tetrachoric matrix (3.233) was almost six times the second (0.558; subsequent values 0.237, 0.163, 0.077), indicating one dominant dimension; parallel analysis on tetrachoric correlations suggested additional minor factors, which is consistent with the heterogeneous content of the index and was not pursued because the one-factor model fitted acceptably (Table S2).

Table S1. Endorsement (% Yes) and tetrachoric correlations of the ten BCEs items (N = 451).

Item	% Yes	1	2	3	4	5	6	7	8	9	10
1 Safe caregiver	87.1	—	.31	.36	.05	.17	.11	.26	.29	.05	.18
2 Good friend	87.1	.31	—	.36	.25	.35	.32	.48	.41	.42	.33
3 Comforting beliefs	78.7	.36	.36	—	.29	.15	.20	.14	.28	.10	.19
4 Liked school	90.9	.05	.25	.29	—	.37	.28	.18	.42	.47	.32
5 Teacher who cared	80.7	.17	.35	.15	.37	—	.38	.31	.29	.27	.31
6 Good neighbors	70.1	.11	.32	.20	.28	.38	—	.38	.48	.54	.45
7 Supportive adult	72.3	.26	.48	.14	.18	.31	.38	—	.51	.24	.23
8 Opportunities to have fun	90.7	.29	.41	.28	.42	.29	.48	.51	—	.57	.33
9 Self-satisfaction	68.3	.05	.42	.10	.47	.27	.54	.24	.57	—	.47
10 Predictable routine	66.3	.18	.33	.19	.32	.31	.45	.23	.33	.47	—

Note. Tetrachoric correlations estimated with the psych package (two-step ML); the mean inter-item tetrachoric correlation was .31 (Pearson phi mean = .16). KR-20 = .655; ordinal alpha (from the tetrachoric matrix) = .816.

S1.2. Confirmatory factor analyses (WLSMV)

One-factor models were fitted to the BCEs items (declared ordered/binary) and to the FSII items (ordered, five categories) with the WLSMV estimator (diagonally weighted least squares with mean- and variance-adjusted test statistic; theta parameterization, std.lv = TRUE). A two-factor model with both scales and a single-factor model with all fifteen items were compared with the Satorra–Bentler scaled difference test. Table S2 reports the fit indices and the standardized loadings with 95% confidence intervals. Model-based reliability (categorical omega) was computed as $(\Sigma\lambda)^2 / [(\Sigma\lambda)^2 + \Sigma(1 - \lambda^2)]$ from the standardized loadings; AVE = mean λ^2.

Table S2. Fit indices and standardized loadings of the confirmatory factor models (WLSMV; N = 451).

Model	Scaled χ^2 (df)	p	CFI	TLI	RMSEA [90% CI]	SRMR
BCEs — one factor	58.04 (35)	.009	.955	.942	.038 [.019, .055]	.082
FSII — one factor	33.94 (5)	< .001	.998	.996	.113 [.079, .151]	.013
Two factors: BCEs + FSII	177.77 (89)	< .001	.993	.992	.047 [.037, .057]	.084
One factor: all 15 items	473.85 (90)	< .001	.971	.967	.097 [.089, .106]	.143

Note. Scaled (mean- and variance-adjusted) indices are reported. The alternative 'robust' indices of Savalei (2018) as implemented in lavaan were lower for the BCEs model (CFI = .867, TLI = .830, RMSEA = .102 [.055, .145]) and similar for the FSII model (CFI = .987, TLI = .974, RMSEA = .122 [.085, .162]); the BCEs fit should therefore be described as acceptable rather than excellent. Two-factor vs. one-factor: scaled $\Delta\chi^2(1) = 82.90$, $p < .001$; latent correlation BCEs–FIS = −.53, 95% CI [−.62, −.44].

Table S2 (continued). Standardized loadings (λ), standard errors, and 95% confidence intervals of the one-factor models.

Scale	Item	λ	SE	z	95% CI	Reliability
BCEs	1 Safe caregiver	.323	.089	3.61	[.148, .498]	ω = .82; AVE = .33
BCEs	2 Good friend	.638	.069	9.19	[.502, .774]	
BCEs	3 Comforting beliefs	.358	.078	4.59	[.205, .511]	
BCEs	4 Liked school	.538	.082	6.52	[.376, .699]	
BCEs	5 Teacher who cared	.506	.073	6.89	[.362, .649]	
BCEs	6 Good neighbors	.679	.055	12.46	[.572, .786]	
BCEs	7 Supportive adult	.543	.064	8.50	[.418, .668]	
BCEs	8 Opportunities to have fun	.745	.072	10.34	[.604, .887]	
BCEs	9 Self–satisfaction	.691	.055	12.62	[.584, .799]	
BCEs	10 Predictable routine	.580	.061	9.48	[.460, .699]	
FSII	1 Thought of hurting oneself	.896	.013	68.84	[.871, .922]	ω = .96; AVE = .83
FSII	2 Life not worth living	.942	.009	109.95	[.925, .959]	
FSII	3 What would happen if life ended	.898	.011	83.64	[.877, .920]	
FSII	4 Thought about suicide	.901	.015	60.87	[.872, .930]	
FSII	5 Wished not to exist	.925	.009	102.49	[.908, .943]	

Note. All loadings $p < .001$. For comparison, the FSII model estimated with robust maximum likelihood (MLR, items treated as continuous) gave ω = .94 and AVE = .76 (CFI = .992, TLI = .983, RMSEA = .085, SRMR = .015). Polychoric correlations among FSII items ranged from .78 to .86.

S1.3. Rasch (one-parameter logistic) model of the BCEs items

A dichotomous Rasch model was estimated by conditional maximum likelihood (eRm::RM). Table S3 reports item difficulties (sum-to-zero constraint; higher values = less frequently endorsed), their standard errors, and infit/outfit mean-square statistics computed from person-parameter estimates. Person-separation reliability was 0.31, which is low, as expected for a ten-item dichotomous index with a pronounced ceiling (23.9% of participants endorsed all items) and reflects the limited capacity of the total score to discriminate among persons in the upper range. The Martin-Löf test did not reject unidimensionality (LR = 31.249, df = 24, p = 0.147). The Andersen likelihood-ratio test was non-significant for the median split (LR = 14.36, df = 8, p = 0.073; item 2 excluded because of a degenerate pattern in one subgroup) but significant for the mean split (LR = 32.37, df = 9, p < .001), indicating some departure from parameter invariance across score levels.

Table S3. Rasch item parameters and fit statistics for the BCEs items (N = 451).

Item	Difficulty (β)	SE	95% CI	Infit MSQ	Outfit MSQ	Infit t	Outfit t
1 Safe caregiver	NaN	−.575	[NaN, NaN]	1.19	0.14	1.24	1.08
2 Good friend	NaN	−.575	[NaN, NaN]	0.69	0.14	−2.27	0.88
3 Comforting beliefs	NaN	.145	[NaN, NaN]	1.23	0.12	2.21	1.13
4 Liked school	NaN	−1.021	[NaN, NaN]	0.86	0.16	−0.66	0.95
5 Teacher who cared	NaN	−.004	[NaN, NaN]	1.04	0.13	0.40	1.01
6 Good neighbors	NaN	.708	[NaN, NaN]	0.90	0.11	−1.46	0.93
7 Supportive adult	NaN	.574	[NaN, NaN]	1.06	0.11	0.76	1.03
8 Opportunities to have fun	NaN	−.991	[NaN, NaN]	0.64	0.16	−2.14	0.81
9 Self−satisfaction	NaN	.812	[NaN, NaN]	0.93	0.11	−1.07	0.95
10 Predictable routine	NaN	.926	[NaN, NaN]	0.98	0.11	−0.33	0.99

Note. MSQ = mean-square fit statistic (values between 0.7 and 1.3 are conventionally regarded as productive for measurement; item 8 shows overfit, outfit = 0.64, and item 3 mild underfit, 1.23). Difficulties are expressed on the logit scale; the easiest experiences to endorse were "I liked school" (item 4) and "opportunities to have fun" (item 8), and the hardest were "predictable daily routine" (item 10) and "self-satisfaction" (item 9).

S1.4. Evidence based on relations with other variables (known groups)

Because no additional established scale was administered, convergent evidence was limited to the FSII and to demographic known-groups comparisons (Table S4). BCEs were negatively correlated with the FIS score (Spearman $\rho = -.322$, $p < .001$) and were markedly lower among students who reported having thought about suicide at least "sometimes" during the previous 12 months (item 4 of the FSII), with a medium effect size, whereas they did not differ by gender, residence area, employment status, marital status, or age group. The FIS score, in turn, showed the expected differences by gender (higher among women) and by age group (lower among older students).

Table S4. Spearman correlations and Mann–Whitney/Kruskal–Wallis comparisons (N = 451).

Comparison	Group 1: M / Mdn	Group 2: M / Mdn	U	p	r
BCEs by gender	men (n = 108): 8.06 / 8.0	women (n = 338): 7.91 / 8.0	18824	.618	.024
FIS by gender	men (n = 108): 8.71 / 6.5	women (n = 338): 10.44 / 10.0	14622	.002	.150
BCEs by area	rural (n = 86): 8.03 / 8.5	urban (n = 365): 7.90 / 8.0	16104	.702	.018
FIS by area	rural (n = 86): 10.10 / 10.0	urban (n = 365): 10.08 / 9.0	15972	.796	.012
BCEs by marital	not single (n = 44): 8.18 / 9.0	single (n = 407): 7.89 / 8.0	10100	.155	.067
FIS by marital	not single (n = 44): 7.09 / 5.0	single (n = 407): 10.41 / 10.0	5463	< .001	.203
BCEs by employment	not working (n = 272): 7.91 / 8.0	working (n = 179): 7.94 / 8.0	24182	.904	.006
FIS by employment	not working (n = 272): 10.61 / 10.0	working (n = 179): 9.30 / 8.0	27954	.007	.127
BCEs by ideation ≥ sometimes (FSII item 4)	< sometimes (n = 347): 8.29 / 9.0	≥ sometimes (n = 104): 6.71 / 7.0	25923	< .001	.324

Note. $r = |z|/\sqrt{N}$ (rank-biserial-type effect size). Kruskal–Wallis by age group: BCEs $H(2) = 2.87$, $p = 0.238$; FIS $H(2) = 35.93$, $p < .001$ (medians 10, 6, and 5 for 16–25, 26–35, and ≥36 years). Spearman correlations: BCEs–FIS $\rho = -.322$; BCEs–age $\rho = .115$ ($p = .015$); FIS–age $\rho = -.313$ ($p < .001$). Item-level Spearman correlations of the BCEs items with FIS ranged from .02 (item 1) to −.40 (item 9).

Section S2. Regression diagnostics

Table S5 summarizes the diagnostics of the four moderation models (BCEs mean-centered). Standardized residuals were moderately right-skewed and platykurtic, and the Shapiro–Wilk test rejected normality in all models, as expected for a bounded outcome with a floor effect (30.2% of participants at the minimum FIS score); with n > 400 the OLS estimates remain unbiased, and inference was checked with HC3 and bootstrap methods (Section S3). The Breusch–Pagan and White tests detected heteroscedasticity in all models. No observation had Cook's distance > 1 or |standardized residual| > 3; between 10 and 21 observations exceeded the conventional 4/n threshold and were removed in the sensitivity analyses of Table S7. Variance inflation factors were below 1.3 in every model, so multicollinearity was not a concern after centering. Figure S3 displays the residual-versus-fitted and normal Q–Q plots.

Table S5. Regression diagnostics for the moderation models (N = 451; N = 446 for the gender model).

Model	Residual skew	Residual kurtosis	Shapiro–Wilk W (p)	Breusch–Pagan χ^2 (df), p	White LM (df), p	Cook's D > 4/n (max)	Leverage > 2p/n	|z| > 3
M1 (age, categorical)	0.49	−0.45	0.967 (< .001)	26.70 (5), < .001	29.39 (2), < .001	10 (max .058)	53	0
M1b (age, continuous)	0.49	−0.45	0.967 (< .001)	19.51 (3), < .001	22.08 (2), < .001	13 (max .051)	35	0
M2 (gender)	0.52	−0.56	0.955 (< .001)	25.91 (3), < .001	27.13 (2), < .001	21 (max .065)	55	0
M3 (marital status)	0.52	−0.53	0.957 (< .001)	21.71 (3), < .001	28.29 (2), < .001	17 (max .157)	57	0

Note. Maximum generalized VIF (adjusted for df) = 1.13 (M1), 1.05 (M1b), 1.28 (M2), 1.14 (M3). In every model the largest |DFBETAS| for the interaction coefficient was below 0.72 and no single observation changed the sign or the significance of any interaction term (see Table S7 for re-estimation without influential cases).

Figure S3. Standardized residuals versus fitted values (top) and normal Q–Q plots (bottom) for the continuous-age, gender, and marital-status models.

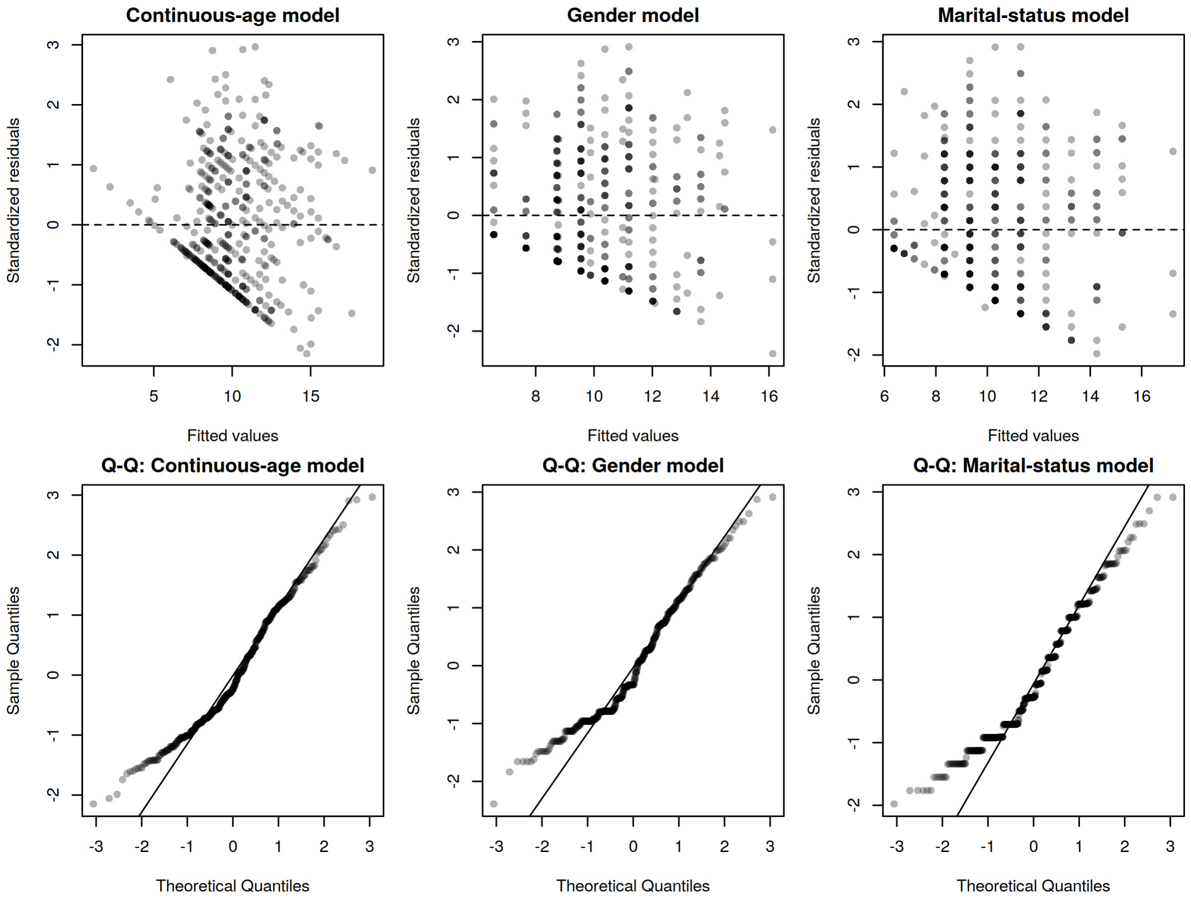
Continuous-age model
Gender model
Marital-status model
Standardized residuals
Fitted values
Q-Q: Continuous-age model
Q-Q: Gender model
Q-Q: Marital-status model
Sample Quantiles
Theoretical Quantiles

Section S3. Heteroscedasticity-consistent and bootstrap confidence intervals; sensitivity analyses

Table S6 reports, for every coefficient of the four models, the OLS estimate with its conventional 95% confidence interval, the HC3 heteroscedasticity-consistent standard error and interval (sandwich package), and the bootstrap percentile and bias-corrected and accelerated (BCa) intervals based on 5,000 case resamples (boot package, seed 2026). Table S7 reports the interaction coefficients re-estimated (a) after excluding observations with Cook's distance > 4/n, (b) with a log-transformed outcome, (c) with MM-type robust regression (MASS::rlm), and, for the age models, (d) after excluding participants older than 50 years and (e) after excluding participants aged 36 years or older.

Table S6. OLS, HC3-robust, and bootstrap estimates for all coefficients of the moderation models.

Model	Coefficient	B (SE)	OLS 95% CI	HC3 SE; 95% CI	Bootstrap percentile 95% CI	Bootstrap BCa 95% CI
M1 categorical age	Intercept	10.629 (.240)	[10.158, 11.100]	.251; [10.137, 11.121]	[10.143, 11.132]	[10.133, 11.127]
	BCEs (centered)	−1.030 (.126)	[−1.277, −.782]	.133; [−1.290, −.768]	[−1.279, −.767]	[−1.277, −.764]
	Age 26–35 (vs. 16–25)	−2.561 (.650)	[−3.839, −1.283]	.547; [−3.636, −1.486]	[−3.616, −1.504]	[−3.550, −1.444]
	Age ≥36 (vs. 16–25)	−4.741 (1.037)	[−6.780, −2.703]	.639; [−5.998, −3.485]	[−5.731, −3.323]	[−5.591, −2.752]
	BCEs × 26–35	.234 (.317)	[−.388, .856]	.274; [−.304, .772]	[−.269, .807]	[−.281, .783]
	BCEs × ≥36	1.167 (.445)	[.292, 2.042]	.176; [.821, 1.513]	[.808, 1.566]	[.846, 1.627]
M1b continuous age	Intercept	10.027 (.216)	[9.602, 10.451]	.215; [9.604, 10.449]	[9.598, 10.467]	[9.621, 10.494]
	BCEs (centered)	−.896 (.111)	[−1.113, −.679]	.110; [−1.112, −.680]	[−1.105, −.672]	[−1.097, −.656]
	Age (centered)	−.234 (.035)	[−.303, −.164]	.028; [−.287, −.180]	[−.291, −.184]	[−.285, −.179]
	BCEs × age	.061 (.018)	[.026, .097]	.014; [.035, .088]	[.034, .088]	[.032, .087]
M2 gender	Intercept	10.437 (.258)	[9.929, 10.945]	.268; [9.910, 10.964]	[9.911, 11.000]	[9.918, 11.008]
	BCEs (centered)	−.822 (.132)	[−1.080, −.563]	.144; [−1.105, −.539]	[−1.098, −.549]	[−1.090, −.536]
	Male (vs. female)	−1.577 (.526)	[−2.611, −.543]	.500; [−2.561, −.593]	[−2.577, −.611]	[−2.541, −.566]
	BCEs × male	−.284 (.282)	[−.838, .269]	.316; [−.905, .336]	[−.864, .361]	[−.876, .353]
M3 marital status	Intercept	10.383 (.233)	[9.924, 10.841]	.239; [9.913, 10.852]	[9.919, 10.856]	[9.915, 10.852]
	BCEs (centered)	−.988 (.120)	[−1.224, −.751]	.125; [−1.233, −.742]	[−1.212, −.746]	[−1.211, −.744]
	Not single (vs. single)	−3.190 (.752)	[−4.668, −1.712]	.631; [−4.430, −1.950]	[−4.282, −1.875]	[−4.263, −1.821]
	BCEs × not single	.594 (.360)	[−.113, 1.302]	.359; [−.112, 1.300]	[−.196, 1.081]	[−.127, 1.125]

Note. M1: R^2 = .200, ΔR^2 (interaction block) = .013, $F(2, 445)$ = 3.54, p = .030. M1b: R^2 = .213, ΔR^2 = .020, $F(1, 447)$ = 11.48, p < .001. M2 (N = 446): R^2 = .136, ΔR^2 = .002, $F(1, 442)$ = 1.02, p = .313. M3: R^2 = .165, ΔR^2 = .005, $F(1, 447)$ = 2.73, p = .100. Bootstrap intervals were computed from 5,000 case resamples.

Table S7. Sensitivity analyses: interaction coefficients B (SE) under alternative specifications.

Model	Interaction	Excluding Cook's D > 4/n	log(FIS) outcome	MM-robust regression (t)	Excluding age > 50	Excluding age ≥ 36
M1 categorical age	BCEs × 26–35	.408 (.349), p = .243	−.000 (.031), p = .993	.270 (.339), t = 0.79	.234 (.318), p = .462	—

Model	Interaction	Excluding Cook's D > 4/n	log(FIS) outcome	MM-robust regression (t)	Excluding age > 50	Excluding age ≥ 36
M1 categorical age	BCEs × ≥36	1.139 (.437), p = .009	.106 (.044), p = .015	1.183 (.477), t = 2.48	1.215 (.462), p = .009	—
M1b continuous age	BCEs × age	.076 (.022), p < .001	.005 (.002), p = .005	.063 (.019), t = 3.25	.058 (.020), p = .003	.055 (.031), p = .078
M2 gender	BCEs × male	−.047 (.348), p = .892	−.029 (.028), p = .296	−.169 (.306), t = −0.55	—	—
M3 marital status	BCEs × not single	.655 (.468), p = .163	.042 (.035), p = .236	.691 (.390), t = 1.77	—	—

Note. Cases retained after excluding influential observations: 441 (M1), 438 (M1b), 425 (M2), 434 (M3). Interaction block of M1 without influential cases: $\Delta R^2 = .013$, $F(2, 435) = 3.80$, $p = .023$; with log(FIS): $\Delta R^2 = .011$, $F(2, 445) = 3.00$, $p = .051$. For the log-transformed models the coefficients are on the log scale. The continuous-age interaction remained significant in every specification except when participants aged 36 or older were excluded (p = .078), which shows that its statistical significance depends partly on the 21 oldest participants; a quadratic age term added to M1b was not significant (p = .112).

Section S4. Continuous-age model, Johnson–Neyman analysis, simple slopes, and sensitivity of the interaction tests

The complete continuous-age model (Table 8 of the main text; BCEs and age mean-centered at 7.92 and 22.12 years) is FIS = 10.03 − 0.896·BCEs − 0.234·Age + 0.061·(BCEs × Age), R^2 = .213. Table S8 reports the conditional slope of BCEs at selected ages with conventional and HC3 95% confidence intervals. The Johnson–Neyman technique located the upper bound of the region of significance at 30.7 years: below that age the slope is significantly negative; between 30.8 and 57.5 years it is not distinguishable from zero; and above 57.6 years (three participants) the point estimate is positive. The bootstrap distribution (5,000 resamples) of the age at which the conditional slope crosses zero had a median of 36.7 years (95% percentile interval 32.0–47.4 years). Figure S1 displays the conditional slope as a function of age with its confidence band and the distribution of ages (rug); Figure S2 displays the predicted FIS values at 18, 25, 35, and 45 years.

Table S8. Conditional slopes of BCEs on FIS at selected ages (continuous-age model), simple slopes by group, and sensitivity of the interaction tests.

Age (years)	Conditional slope b	95% CI (OLS)	95% CI (HC3)	t
16	−1.271	[−1.581, −.960]	[−1.573, −.968]	−8.04
18	−1.148	[−1.413, −.883]	[−1.414, −.882]	−8.53
20	−1.026	[−1.257, −.794]	[−1.263, −.789]	−8.71
22	−.903	[−1.121, −.686]	[−1.120, −.687]	−8.17
25	−.720	[−.958, −.482]	[−.929, −.511]	−5.95
30	−.414	[−.764, −.064]	[−.673, −.155]	−2.32
35	−.108	[−.610, .393]	[−.463, .247]	−0.42
40	.198	[−.468, .864]	[−.271, .667]	0.58
45	.504	[−.332, 1.339]	[−.088, 1.095]	1.18
50	.810	[−.198, 1.818]	[.092, 1.527]	1.58
60	1.422	[.065, 2.778]	[.446, 2.397]	2.06

Moderator	Group	Simple slope b (SE)	95% CI (OLS)	95% CI (HC3)	p
Age group (M1)	16–25	−1.029 (.126)	[−1.277, −.782]	[−1.290, −.768]	< .001
Age group (M1)	26–35	−.795 (.290)	[−1.366, −.225]	[−1.266, −.325]	.006
Age group (M1)	≥36	.138 (.427)	[−.702, .977]	[−.090, .365]	.747
Gender (M2)	women	−.822 (.131)	[−1.080, −.563]	[−1.104, −.539]	< .001
Gender (M2)	men	−1.106 (.249)	[−1.596, −.617]	[−1.658, −.554]	< .001
Marital status (M3)	single	−.987 (.121)	[−1.224, −.751]	[−1.233, −.742]	< .001
Marital status (M3)	not single	−.393 (.339)	[−1.060, .273]	[−1.055, .268]	.247

Interaction test	Minimum detectable f^2 (80% power, α = .05)	≈ ΔR^2 detectable	Observed f^2	Post hoc power for observed f^2
BCEs × age group (2 df)	.021	.017	.016	0.66
BCEs × age (continuous)	.018	.014	.026	0.93
BCEs × gender	.018	.015	.002	0.17
BCEs × marital status	.018	.015	.006	0.38

Note. Sensitivity analysis: the minimum detectable effect size was obtained by solving the non-central F distribution for 80% power with the numerator df of the interaction term and the residual df of the model. Post hoc power for the observed f^2 is reported only as a descriptive complement. The gender and marital-status interaction tests were underpowered for effects smaller than f^2 ≈ .018 (ΔR^2 ≈ .015), so their non-significance does not establish equivalence across groups.

Figure S1. Conditional slope of BCEs on suicidal ideation as a function of age (Johnson–Neyman plot). The shaded band is the 95% confidence interval; the dotted vertical line marks the upper bound of the region of significance (30.7 years); rug marks show the observed ages.

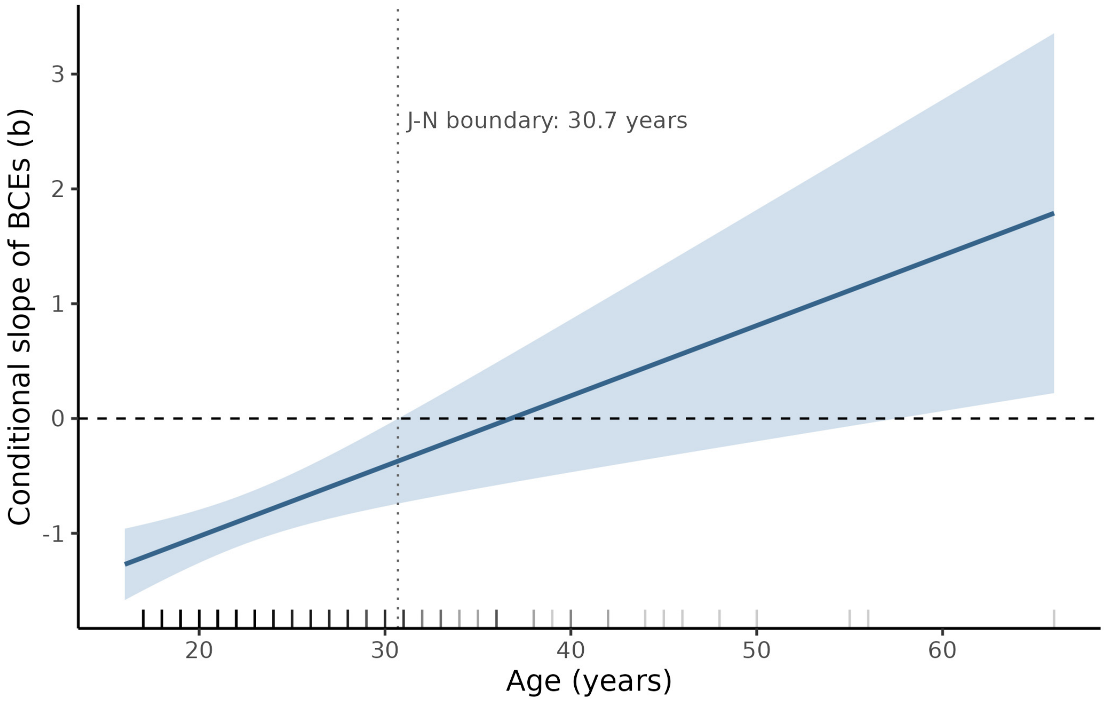

Figure S2. Predicted frequency of suicidal ideation as a function of BCEs at 18, 25, 35, and 45 years (continuous-age model).

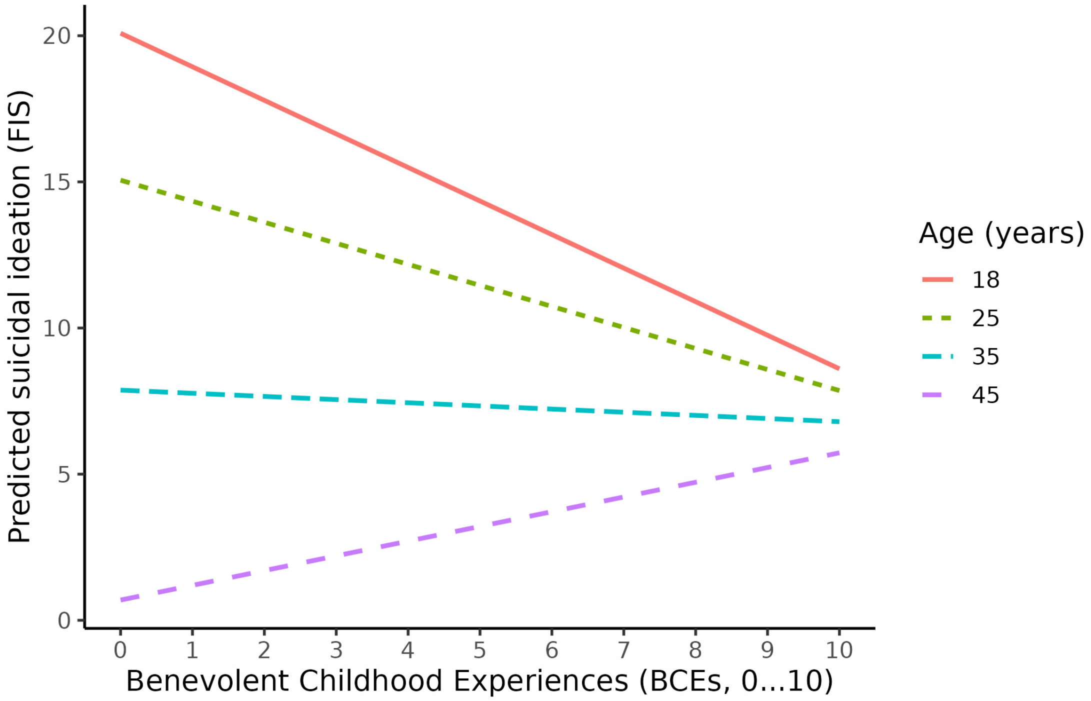

Section S5. Additional sensitivity analyses: covariate adjustment, alternative coding of marital status, linearity, and multiple testing

Table S9 reports four sets of analyses added in response to the reviewers. (a) Each moderation model was re-estimated adjusting for the covariates available in the dataset—university (César Vallejo University vs. other), field of study (psychology vs. other), residence area (rural vs. urban), and employment status (working vs. not working); no measures of depressive symptoms, anxiety, adverse childhood experiences, current distress, substance use, socioeconomic status, or social support were collected, so adjustment for these confounders was not possible. (b) Because the non-single category combines heterogeneous situations, the marital-status model was re-estimated comparing partnered students (cohabiting or married) with single students after excluding the three divorced or widowed participants, and the cohabiting and married subgroups were examined separately. (c) Linearity of the BCEs–FIS relation was checked by adding a quadratic BCEs term to each model and with the Ramsey RESET test (powers 2–3 of the fitted values). (d) Because three moderation hypotheses were tested, Bonferroni- and Holm-adjusted p-values are reported for the interaction tests (family-wise $\alpha = .017$).

Table S9. Additional sensitivity analyses (N = 451; N = 446 for the gender model).

(a) Covariate-adjusted models	Interaction term	B (SE)	95% CI	p	Unadjusted B	R^2 of adjusted model
Age, categorical	BCEs × 26–35	0.231 (0.317)	[−0.392, 0.853]	.467	0.234	.208
	BCEs × ≥36	1.236 (0.448)	[0.356, 2.116]	.006	1.167	
Age, continuous	BCEs × age	0.061 (0.018)	[0.025, 0.096]	< .001	0.061	.219
Gender	BCEs × male	−0.276 (0.279)	[−0.825, 0.273]	.323	−0.284	.161
Marital status	BCEs × not single	0.533 (0.359)	[−0.173, 1.239]	.139	0.594	.181

Note. Covariates: university, field of study, residence area, employment status. In the adjusted models the main association of BCEs was unchanged (b from −0.82 to −1.03, all p < .001); psychology students reported somewhat higher FIS scores (b ≈ 0.9–1.2, p between .02 and .08) and working students somewhat lower scores in the gender model (b = −1.07, p = .023); university and residence area were unrelated to FIS.

(b) Alternative coding of marital status	n (group)	Interaction B (SE)	95% CI	p	Slope of BCEs in the group
Partnered (cohabiting or married) vs. single; divorced/widowed excluded	41 vs. 407	0.556 (0.373)	[−0.178, 1.290]	.137	−0.43
Cohabiting vs. single	23 vs. 407	0.314 (0.518)	[−0.704, 1.332]	.544	−0.67
Married vs. single	18 vs. 407	0.793 (0.522)	[−0.233, 1.819]	.129	−0.19
Not single vs. single, adjusted for covariates (a)	44 vs. 407	0.533 (0.359)	[−0.173, 1.239]	.139	—

Note. Mean age: single 21.2 years; cohabiting 26.9; married 33.8; divorced 34.5; widowed 50.0. Confidence intervals for the subgroup rows were computed as B ± 1.96 SE.

(c) Linearity	Quadratic BCEs term B (p)	RESET F (p)
Age, categorical	0.047 (.301)	—
Age, continuous	0.031 (.494)	1.73 (.179)
Gender	−0.011 (.811)	1.43 (.242)
Marital status	0.012 (.794)	0.28 (.757)

(d) Multiple testing (three moderation tests)	Unadjusted p	Bonferroni-adjusted p	Holm-adjusted p	Meets α = .017?
BCEs × age group (categorical, 2 df)	.030	.090	.090	No
BCEs × age (continuous)	< .001	.002	.002	Yes
BCEs × gender	.313	.940	.313	No
BCEs × marital status	.100	.299	.199	No

Note. Holm-adjusted values are shown separately for the family that includes the categorical age test and for the family that includes the continuous age test (gender and marital status adjusted accordingly). No adjustment was planned a priori; the adjusted values are reported for transparency, and the age moderation is presented as exploratory. Post hoc sensitivity for the main association: with N = 451 the study had 80% power (two-sided α = .05) to detect a correlation of $|r| \geq .13$.
